# Supplementary figures and images for: Citrate serves as a signal molecule to modulate carbon metabolism and iron homeostasis in Staphylococcus aureus
Source: PLoS Pathog. 2024 Jul 30;20(7):e1012425. doi: 10.1371/journal.ppat.1012425 (PMC11315280; doi:10.1371/journal.ppat.1012425)

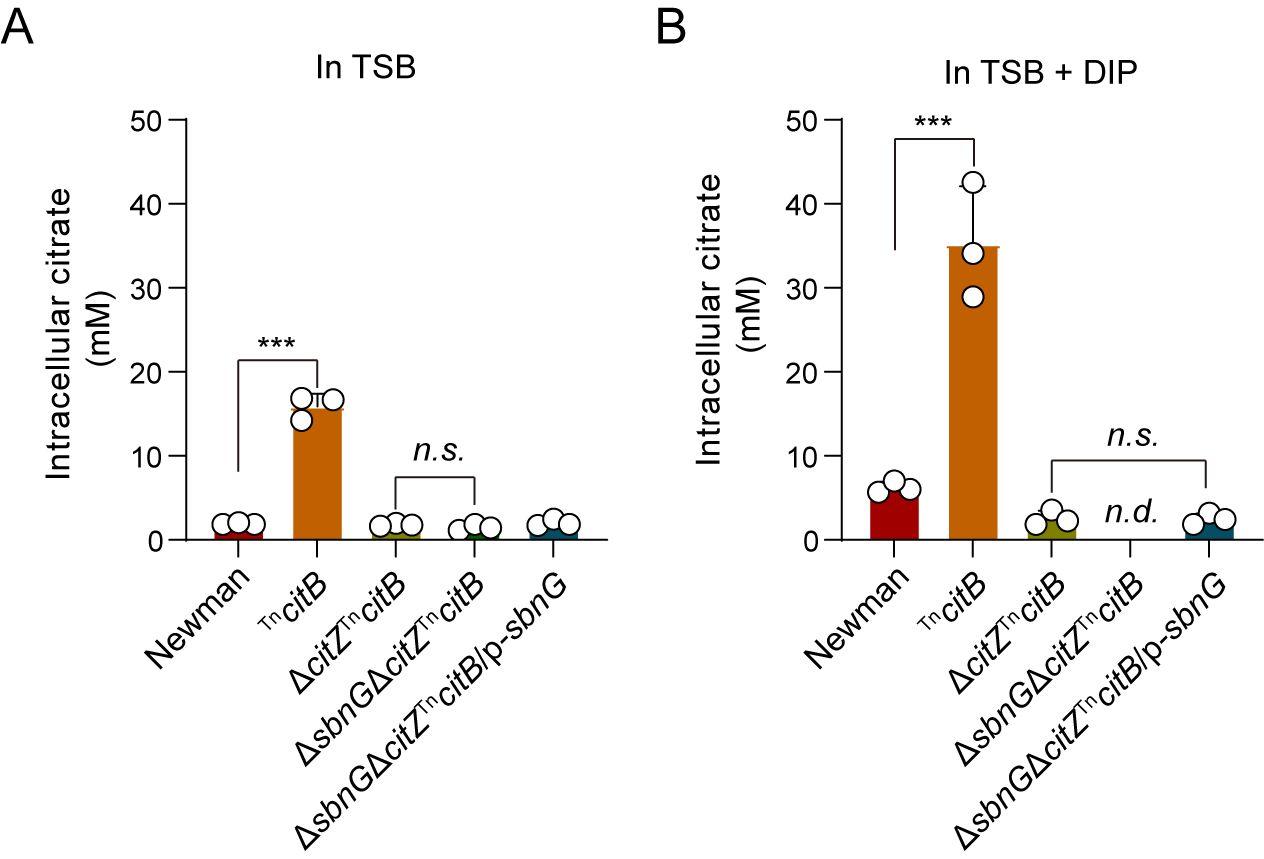

Supplement: S1 Fig — The intracellular citrate levels of S. aureus Newman and its isogenic mutants cultured either in TSB for 6 h (A) or in TSB with 1 mM 2,2-dipyridyl (DIP) for 2 h (B), measured by citrate assay kit. Newman, TncitB, ΔcitZTncitB and ΔsbnGΔcitZTncitB harbor an empty control vector, respectively. ΔsbnGΔcitZTncitB/p-sbnG represents the ΔsbnGΔcitZTncitB strain complemented with sbnG. Data represents mean ± SD from n = 3 biological replicates, n.s., p > 0.05; *** p < 0.001 by One-way ANOVA with Tukey test. n.d., not detectable. (TIF) [file ppat.1012425.s001.tif]

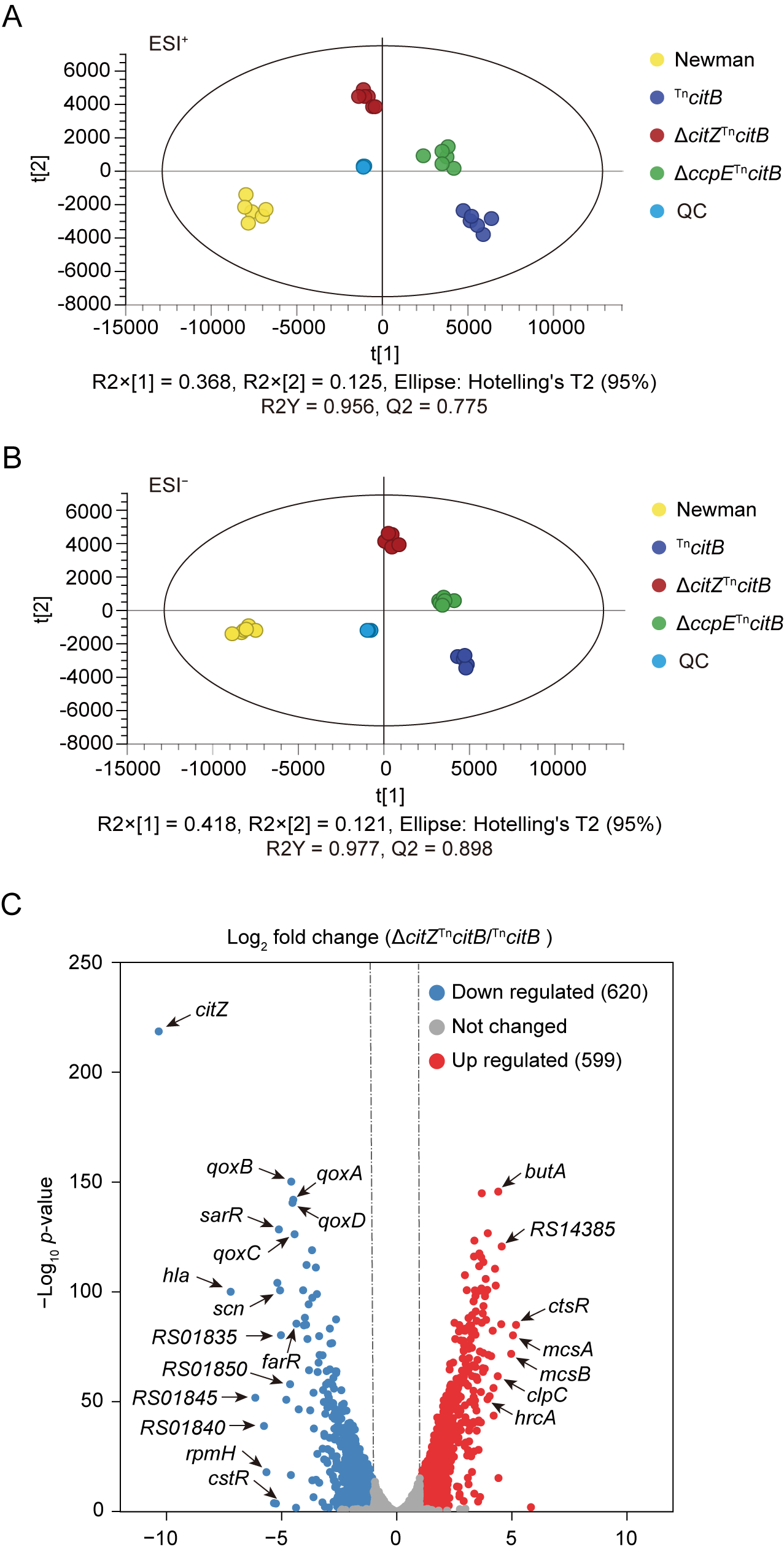

Supplement: S2 Fig — (A and B) Orthogonal partial least squares-discriminant analysis (OPLS-DA) of electrospray ionization (ESI) features generated from ultra-performance liquid chromatography-coupled-quadrupole time of flight mass spectrometry (UPLC-Q-TOF-MS/MS) in positive (A) and negative (B) ionization mode. Data represents n = 6 biological replicates for Newman, TncitB, ΔcitZTncitB and ΔccpETncitB, as well as n = 3 for the pooled quality control (QC) samples. (C) (DEGs) between ΔcitZTncitB and TncitB mutants. X-axis and y-axis represent log2 fold-change differences and statistical significance (present as the negative log of DEG p-values), respectively. Significantly up-regulated and down-regulated genes are indicated by red and blue dots, while non-significant genes are shown as grey dots. (TIF) [file ppat.1012425.s002.tif]

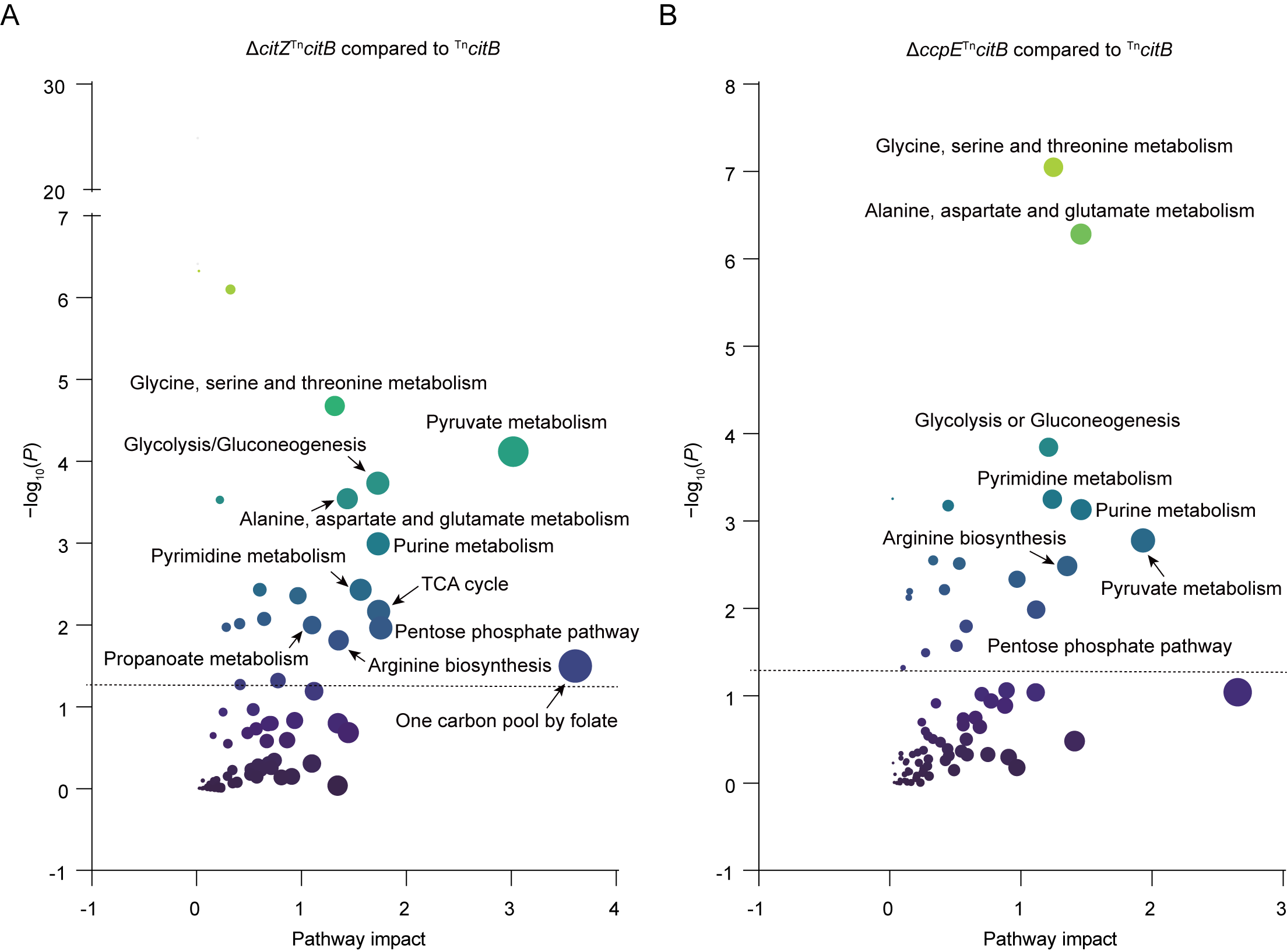

Supplement: S3 Fig — (A) Identification of the citrate-regulated pathway through transcriptomics-metabolomics coupled analysis. (B) Identification of CcpE-regulated pathway through transcriptomics-metabolomics coupled analysis. The x axis represents pathway impact scores, which summarize normalized topology measures of the perturbed genes/metabolites within each pathway. The y axis displays the −log10(P) values derived from the enrichment analysis results. The size of each data points is proportional to its x-axis values, and the color gradients correspond to their y values. The dashed line signifies a significance threshold, corresponding to a p value equal to 0.05, which equates to a -log10(P) of 1.30. (TIF) [file ppat.1012425.s003.tif]

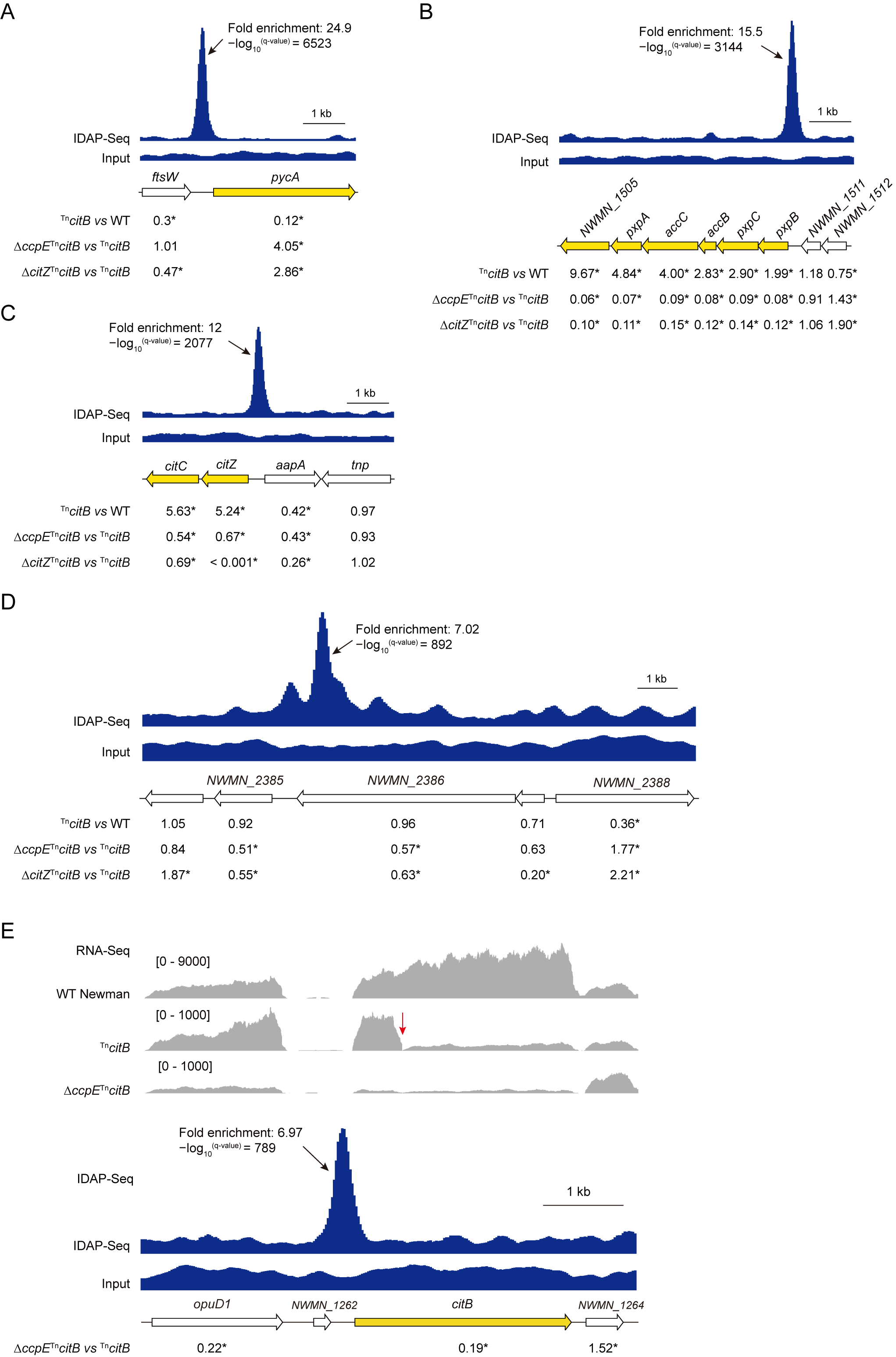

Supplement: S4 Fig — Images represent the representative of CcpE IDAP-seq data showing the binding of CcpE to the promoter region of pycA (A), the promoter region of pxpB (B), the promoter region of citZ (C), the NWMN_2386 open reading frame region (D), and the promoter region of citB (E). IDAP-seq data originated from replicate 1 (Biosample accession number: SAMN24812582) was used to generate the images with Integrated Genome Viewer (IGV) software. The fold enrichment and -log10(q-value) of the IDAP-seq peaks produced by MACS2 were presented. The ORF arrows below the IGV images indicate the direction of transcription (left or right). The gene names (or locus tag) are indicated above the ORF arrows, while the relative expression (fold-change) is indicated below the ORF (*p < 0.05 by edgeR exact test). Potential CcpE-targeted genes are highlighted by yellow arrows. The IVG visualization of RNA-seq data for citB loci is also presented in (E), and the transposon insertion site of the TncitB mutant is marked by a red arrow. (TIF) [file ppat.1012425.s004.tif]

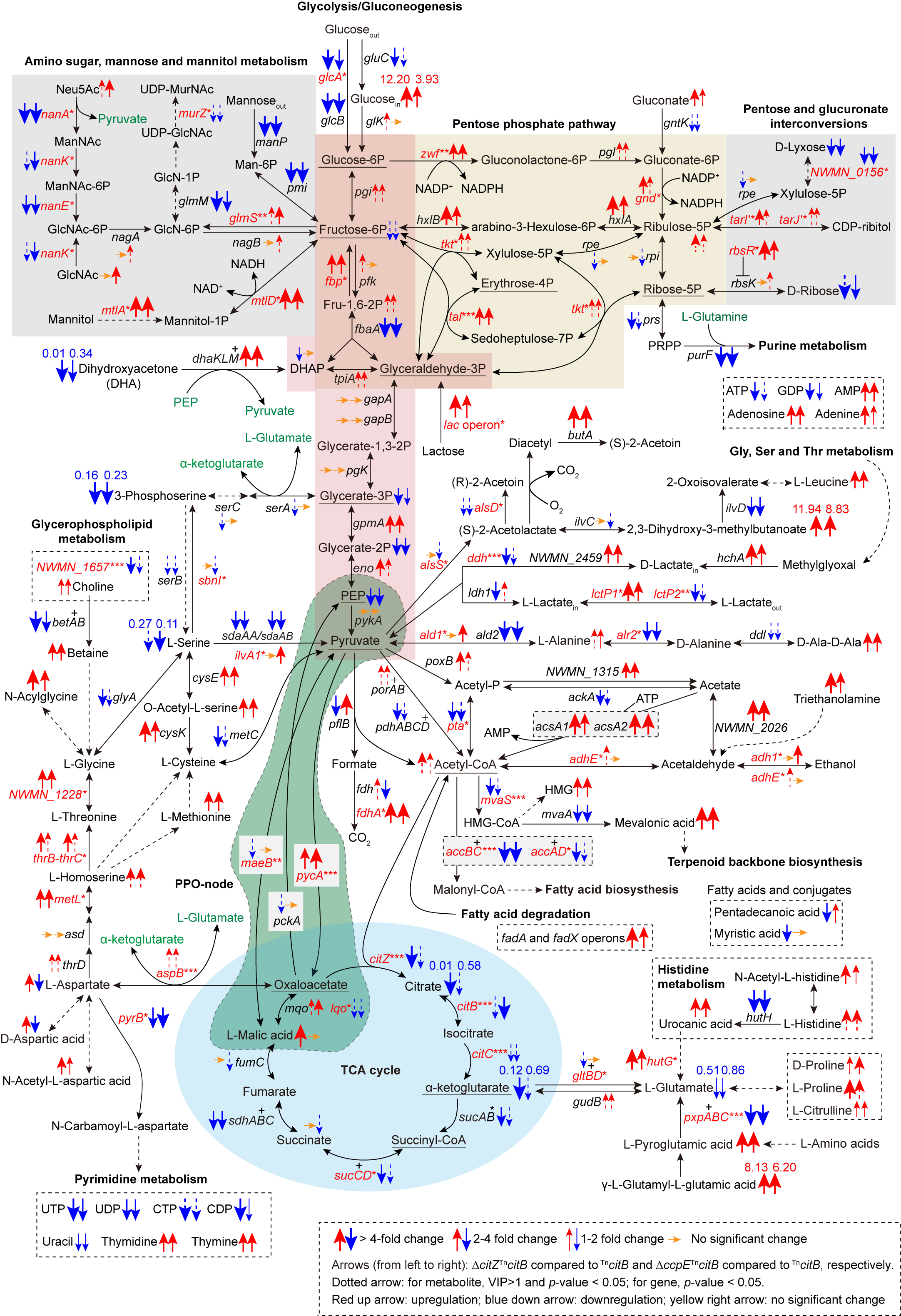

Supplement: S5 Fig — The pink shading indicates glycolysis/gluconeogenesis, the light-yellow shading indicates the pentose phosphate pathway, the blue shading indicates the TCA cycle, and the green shading indicates the phosphoenolpyruvate-pyruvate-oxaloacetate (PPO) node. The arrows, from left to right, respectively indicated ΔcitZTncitB/TncitB and ΔccpETncitB/TncitB. The upwards arrow in red, denotes upregulation, and the downwards arrow in blue, denotes downregulation. The 12 well-known branchpoint metabolites that serve as exit points for carbon from the central carbon metabolism network are underlined [32]. The direct reactions are indicated by continuous line with an arrow at the end, while multiple steps of reaction are indicated by dashed lines with an arrow at the end. The genes of interest are notated in italics. The asterisks (*, **, and ***) indicates a gene or operon whose promoter region was bound by CcpE in one, two, and three independent IDAP-seq experiments, respectively. PEP, phosphoenolpyruvate; CoA, coenzyme A; PRPP, 5-Phosphoribosyl diphosphate; Fru-1,6-2P, fructose 1,6-bisphosphate; DHAP, dihydroxyacetone; GlcN-6P, Glucosamine 6-phosphate; GlcN-1P, Glucosamine 1-phosphate; GlcNAc, N-Acetyl-glucosamine; GlcNAc-6P, N-Acetyl-glucosamine 6-phosphate; UDP-GlcNAc, UDP-N-Acetyl-glucosamine; UDP-MurNAc, UDP-N-Acetylmuramic acid; Man-6P, Mannose 6-phosphate; ManNAc, N-Acetyl-mannosamine; ManNAc-6P, N-Acetyl-mannosamine 6-phosphate; Neu5Ac, N-Acetylneuraminate. (TIF) [file ppat.1012425.s005.tif]

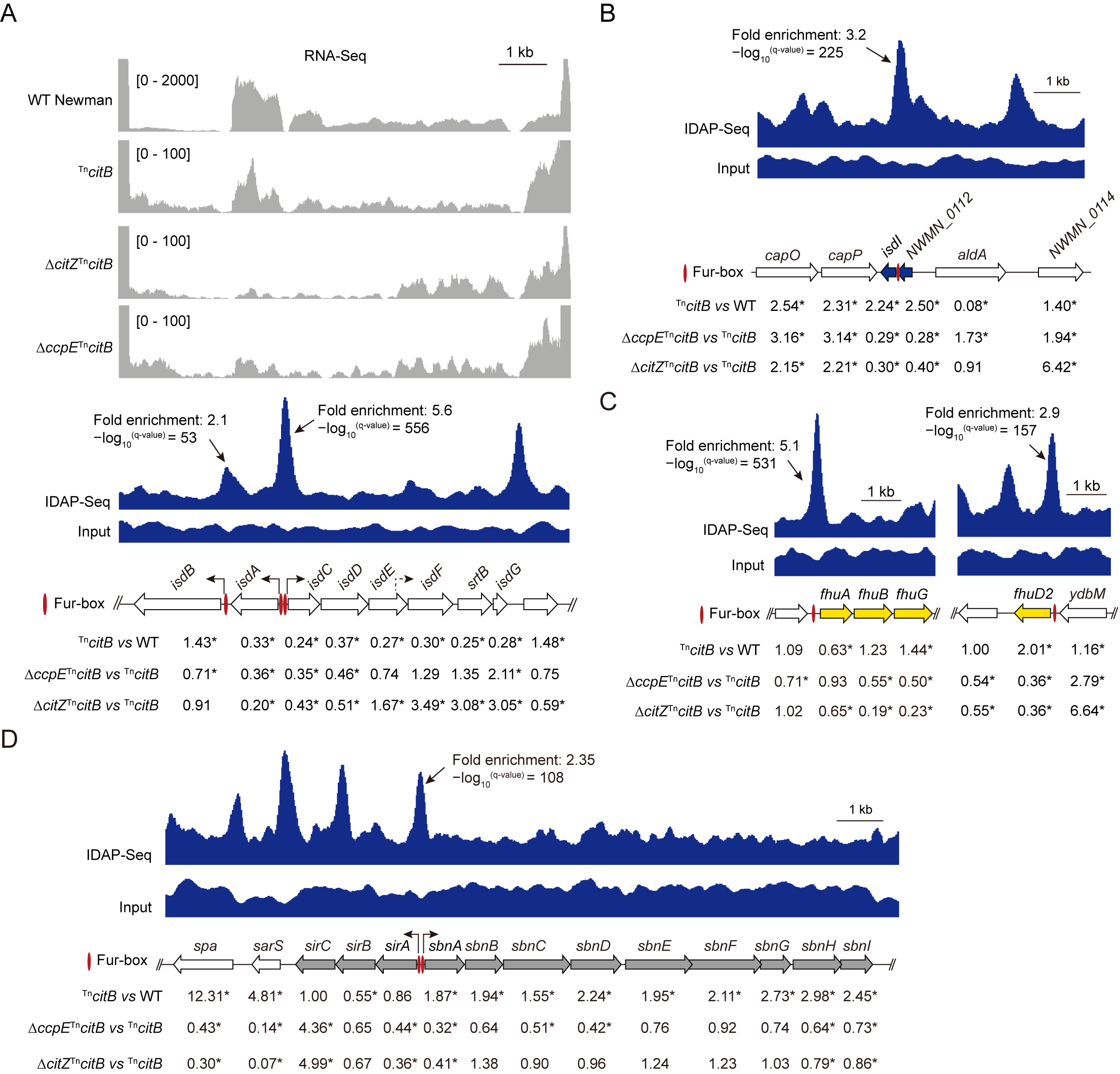

Supplement: S6 Fig — Representative images of CcpE IDAP-seq data showing the binding of CcpE to the promoter region of isdB (in A), the intergenic region between isdA and isdC (in A), the promoter region of isdI (in B), the promoter region of fhuABG operon and fhuD2 (in C), and the intergenic region between sirA and sbnA (in D). IDAP-seq data originated from replicate 1 (Biosample accession number: SAMN24812582) was used to generate the images with Integrated Genome Viewer (IGV) software. The fold enrichment and -log10(q-value) of the IDAP-seq peaks generated from MACS2 were presented. Arrows below the IGV images represent ORFs pointing left or right, indicating the direction of transcription. Gene names (or locus tag) are shown above the ORF arrows. Numbers below the ORF are relative expression levels (fold-change) (*p < 0.05 by edgeR exact test). The IVG visualization of RNA-seq data for the isd loci, as depicted in (A), reveals the presence of a potential internal promoter situated upstream of the isdF-srtB-isdG. This internal promoter may account for the observed differential expression pattern of the isd operon genes. (TIF) [file ppat.1012425.s006.tif]

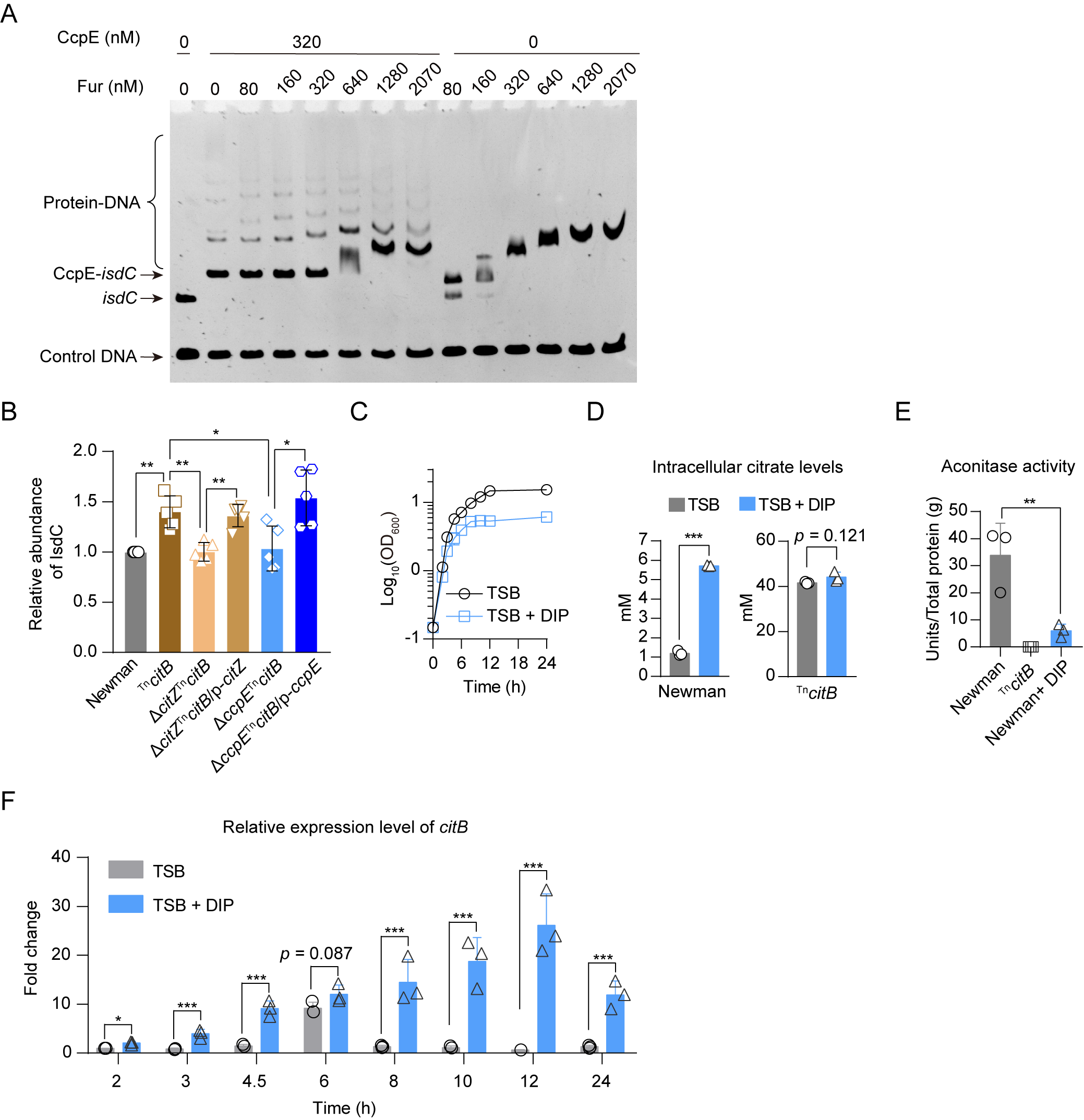

Supplement: S7 Fig — (A) EMSA result shows that Fur can compete CcpE for binding to the isdC promoter region. (B) Quantitative analysis of Western blotting for IsdC in Fig 4E. Glyceraldehyde 3-phosphate dehydrogenase (GAPDH) was used as the loading control. The results are reported as relative abundance with the control sample (Newman) set to 1. (C) Growth curve of S. aureus Newman in TSB without or with 1 mM DIP. (D) Intracellular citrate levels of S. aureus strains cultured in TSB without or with DIP for 2 h. (E) Aconitase activities of WT Newman and TncitB mutant. (F) RT-qPCR analysis of citB transcripts in WT S. aureus Newman strain grown in TSB without or with 1 mM DIP. Results were reported as fold changes with the 2 h (without DIP) group set to 1. In (B), data represents mean ± SD from n = 5 biological replicates. In (C to F), data represents mean ± SD from n = 3 replicates. * p < 0.05, **p < 0.01, ***p < 0.001, by two-tailed one-sample t-test when compared with the Newman control where set to 1 (B and F), otherwise two-tailed unpaired t-test was used. (TIF) [file ppat.1012425.s007.tif]

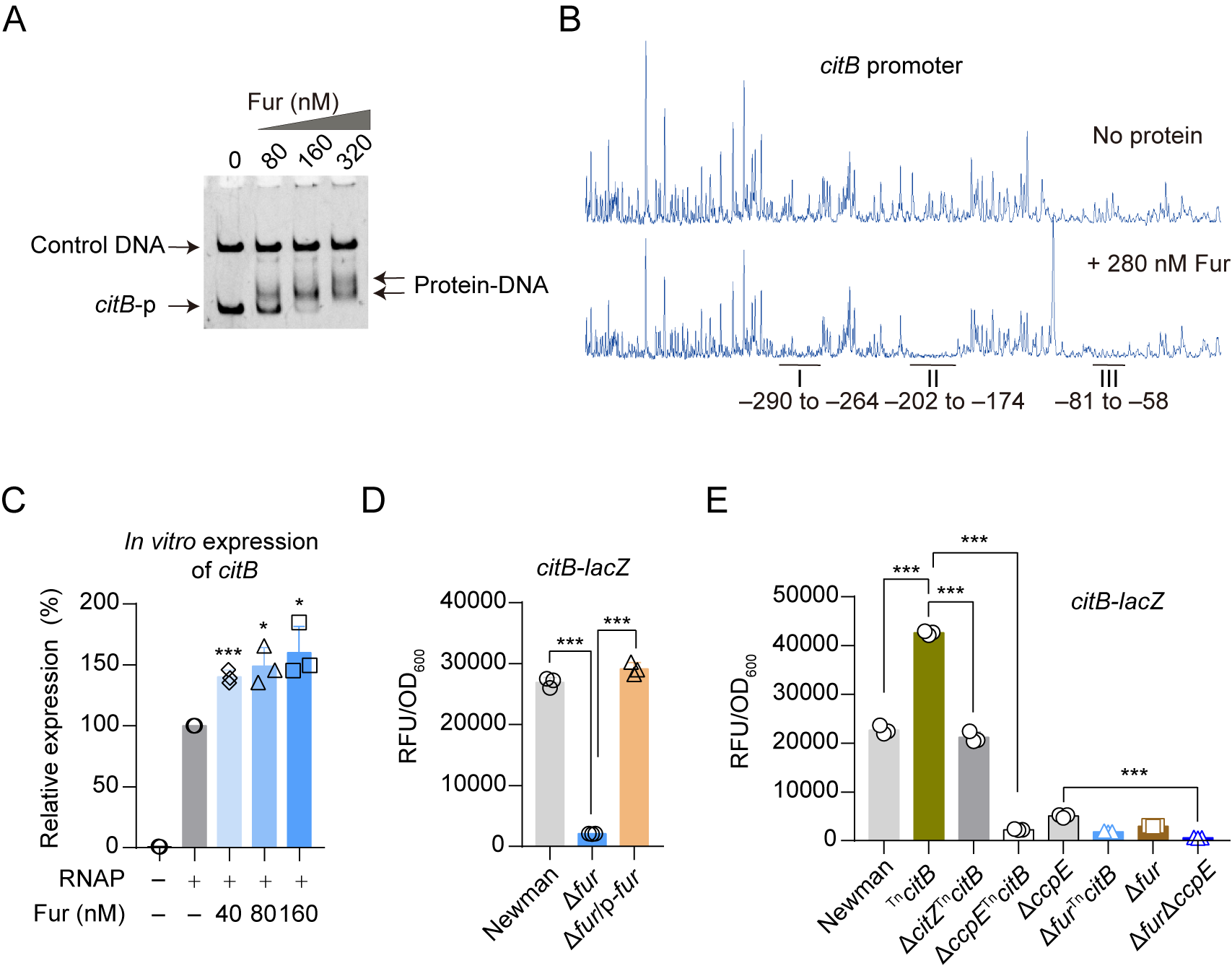

Supplement: S8 Fig — (A) EMSA shows that Fur binds to citB promoter in vitro in a concentration dependent manner. The citB-p fragment contains a DNA sequence covering nucleotide -624 to nucleotide +73 relative to the start codon of citB. An DNA fragment containing the coding region of murA gene was served as control. (B) The protection pattern of the citB promoter after digestion with DNase I following incubation in the absence and presence of Fur in vitro. The protected regions (relative to the start codon) are underlined. (C) Fur enhances citB mRNA expression in vitro. The transcripts were extracted and quantified by RT-qPCR. Data represents mean ± SD from n = 3 independent experiments. Statistical analysis was performed using two-tailed one-sample t-test compared with the RNAP group, which was set to 100% (*p < 0.05, ***p < 0.001). (D and E) citB-lacZ activity in S. aureus strains grown in TSB for 4.5 h. The relative fluorescence intensity (RFU) was normalized to the optical density of bacteria at 600 nm (OD600). Data represents mean ± SD from n = 3 biological replicates and statistical analysis was performed using Student’s two-tailed unpaired t-test (***p < 0.001). (TIF) [file ppat.1012425.s008.tif]
