# Supplementary material for: Citrate serves as a signal molecule to modulate carbon metabolism and iron homeostasis in Staphylococcus aureus
Source: PLoS Pathog. 2024 Jul 30;20(7):e1012425. doi: 10.1371/journal.ppat.1012425 (PMC11315280; doi:10.1371/journal.ppat.1012425)
Supplement: S4 Table — (DOCX) [file ppat.1012425.s012.docx]

**S4 Table. Sequences used for MEME analysis of the binding motif for CcpE**

| No. | Peak no. in IDAP-seq | enrichment fold | Location in Genome of Newman | Sequences^#^ |
| --- | --- | --- | --- | --- |
| 1 | 149*** | 24.87 | 1085205-1085285 | TGTGGTGGCGAAATTAGAATCATGATGATAAGATAAAGTTATCGCTATTGATAATAAATGTTGATGTTTTGCAATTTTATT |
| 2 | 230*** | 15.47 | 1676453-1676533 | TTCCGAGAAAACAAATTGATTATCATTTGTTATTGTATGCGATTTGAATTACTTATTAAACAACTTGTGCTTCTCAATGAT |
| 3 | 237*** | 12.01 | 1760924-1761004 | TGTGTAAAATTCCATGTTAATATACTTTGGTTTTTATAATCATATATAATAACCAATTGAAAATTTAATTCTATTGTAAAA |
| 4 | 364*** | 7.02 | 2620049-2620129 | ACATGATAAAAAGCCAAGAAATGCCAATATTTATCACTTATGATAAGCATGAGGATATAAGTGATAGCACAAAATATGAAG |
| 5 | 193*** | 6.96 | 1386929-1387009 | ATTCTCAATTAATAAAAAATACTTATTACACCTTACCTGTATGATAAGTTTTGCTTATATACTCTGATTAAAAAGTCAAAA |
| 6 | 33*** | 6.01 | 244980-245060 | CATTATATCAGACGTTTTCATACACAAATAATAACATACAAGCAAACATTTCGTTTATTATTTATATCACTTAACTAATTA |
| 7 | 112*** | 5.76 | 856716-856796 | GTTATTTTATTATAATTATTAAATTTTTATTAATTAATTGTAAAAATGTAGAATTATAATTAATTAACGTTTAATATTAAA |
| 8 | 375*** | 5.75 | 2705163-2705243 | TCTGAGATAGGCTAGTGATTGGCTCTATAATGTCGCGGTTTACAGTTGGATCTTCGCTCCAACTGCATAAGAGCCACTAAT |
| 9 | 155*** | 5.56 | 1145369-1145449 | TTTTCGTATAACAATAATAAATTGATTATAAAATAAATATTGACAATGATAATCATTATTATTTATGATTTTACTAAAGAC |
| 10 | 136*** | 5.53 | 1006241-1006321 | TTCAAAATTAGATGCCATTTGCTGTTTATAATAAGAAGATTTGATAATTAACTATGATAAGAAAAAATAATAATCCCCTAA |
| 11 | 7*** | 5.37 | 55365-55445 | GAAATTTTTGATAATATTAAAGTGAAAAAGTGTTATAAATTGATAAATATATGTAATTAACAAAAACAAATCATTTTAAAA |
| 12 | 371*** | 5.20 | 2665271-2665351 | ATAACACTAAATTTCCCAAAATTCAAAAGTGTGTTTTATTGCAGAAAACTTATAACATGTGCACAAGTTATAGTGAATTGC |
| 13 | 84*** | 5.14 | 698339-698419 | TCAAAGACATCCCAATTGATAATGATTATCATGAACATCATTATAACATTTTTCAATCTTATTGACTAACATTACTTTTTA |
| 14 | 333*** | 5.08 | 2439418-2439498 | TTGGCTATTTAACTTTAATAAGCTAGGGCCAAGTTTGTCATAAGATAAGCGCACATTATAATCATACTATTTTGCTTTTAC |
| 15 | 253*** | 4.99 | 1863348-1863428 | AAAAACACTAATGATTTATTATGTAGTGGTTCTTTATCATTAGCTATAACTAATGTGTACTTAAAAATAGGAATACATGAG |
| 16 | 258*** | 4.97 | 1890069-1890149 | TTTGTGACACTTTTAATAGTGTAACCATTGATTATTACAATTTATCAAATGGTCCTTTAGAAGGTATAAATAACAAAATAA |
| 17 | 286*** | 4.95 | 2083337-2083417 | ATTTTATTTATTATTGGTTATTATTTACAAGCAACTAATCATGAAAGCCAAGGTATAAAATTATTATTAGCAGCGATTATG |
| 18 | 142*** | 4.91 | 1027481-1027561 | CTTACAAAAATTAACTTATCATTCATTTATGCTTTACGCTACATAGTAATAAGTTTTTCTAATATGTTATCTATACAACAA |
| 19 | 5*** | 4.82 | 48224-48304 | TTTTTATGATAACTATATCACAATTGGATAATATGAGATTTTACTTATTATTGCTATCCTTTTTATAAAAGGGGGTATGCT |
| 20 | 49*** | 4.72 | 432052-432132 | GTATAAGCAAAATAAATGATTCTGATCCTACTGGAACGATAATAATTTCAGAAGATGACATGAATGAAATCGAAAAGCAAT |
| 21 | 259*** | 4.70 | 1899008-1899088 | TGAAAGCTACAACATTTCTATAAAATTTTTCAATAACAATTGCGCCACTAAAACTCAAAATTTCCACCACCAACATCCAAA |
| 22 | 271*** | 4.67 | 1975148-1975228 | TTTAGTTCCTGATGATGATAACCGTTTCGTTGCATCCGTTACACAACAACCGATACAATTACTTTTCAATGATAATAATCA |
| 23 | 55*** | 4.48 | 481113-481193 | AGGGGTAATTATAAAATTTGATGATACAGTATATGATTTTTTTGTAATCATAATGTCATCAAACATCAACCTATTATACAT |
| 24 | 252*** | 4.45 | 1854173-1854253 | TTATTGTTGTCCATATTATTATATATAAATGAAATCAACATCAATAATAGTGTAATTATACATAATTATTTTTGATTGTTT |
| 25 | 85** | 4.35 | 705466-705546 | TCAGCAAATCCTACGAAACTTAATAAATAACCTGCTACCATCATTATAATAATAAAGATATGATAATTTTTACTGTATGGT |
| 26 | 147*** | 4.32 | 1075338-1075418 | TAATATCAGCAATTGTAACGATTCCTTTAGTTTTACTTATCGTTACTTTAATCATTCCGATATTATTCTTTATTATTGCGA |
| 27 | 272** | 4.30 | 1981299-1981379 | ATATCATTTTTATTAGTAATAAAACCTAATTCAGATAAACGATAATTTATATTTATTTCTGCTGATACATTAACATTTAGT |
| 28 | 47** | 4.27 | 390430-390510 | ACATTTTAATTATTTCATAAAATTTTATTGATAATAATTATCGTTCGGTATAAAGTAAATACTATATACTACTTATGAGTG |
| 29 | 119*** | 4.24 | 880762-880842 | TAATTAATATGCATTTTTAGTCATAGTTACTTTAATGCATTCATTAGTAAAATGATGCTATAGTTCGAGCTGTTTGTTATT |
| 30 | 156*** | 4.22 | 1150463-1150543 | AGTTAATATTAATTAATCGCTTTTATCACTCATAATATTTCAAATTGTATAAATTTCTTTTATCGATACTACTACTATAAA |
| 31 | 113*** | 4.22 | 860309-860389 | TGAGTATCACACCACAAATGATAATACCAGGCAGGAGCCAACATAAATCATCTAAATCTTTATTATATGTGATTAAAATAT |
| 32 | 125** | 4.11 | 932373-932453 | GTCTTACTTATTTATTTGTTATTTGATAACGAAAAAAGTTATAATGTGAATTAAGATAAAGATGAGGAGTTGAGAATGAAT |
| 33 | 178*** | 4.10 | 1326309-1326389 | GGTATTCTTCAAAGGTTTATAAAATAATTTTATGATATATGATTTCAAACAGAGTCATTGTTCATTTTAATATTGAACGCC |
| 34 | 383*** | 4.07 | 2792774-2792854 | CAAAACTGTTATGTATTTATAAATATAATCATTAGTATTTTTATGGCTGAAAAAGTTATAATAAAAGTGTAAGGAATAAAA |
| 35 | 26*** | 4.05 | 198138-198218 | TTATCGTAGCGATCTTGATAACAAGAGCATACAACTTTAATGATTATCATATATGATACAAAATTCTCAATATAAAAAGAA |
| 36 | 358*** | 4.04 | 2566928-2567008 | AATATAAGAAAAAACATTTGATAGGTAAGGGTATGATAACTAAAACTTATCGATTTTTTGAAAAGATATAAGTTTTTATGC |
| 37 | 183*** | 4.03 | 1351285-1351365 | TAATGGTAAAATGACGATGAAATTGAATAATGTTAAGCAAAAGCGTCATATTTTATGCACAAATGAATATAATAATAAGAA |
| 38 | 150*** | 4.02 | 1089228-1089308 | GTTGCAGAAGTTGAATCTCAAACTGAAGTAACGGAATAACAAGTATATAACAGAGGCTAATGCTTTAGCCTCTTGTTATTT |
| 39 | 330*** | 4.01 | 2423629-2423709 | TATATATAACCTTAAATTAACCTATTTAAACGCCGACGTCCACCAATTATTTTTATTTTTAATAATTGTTTAATAAACATG |
| 40 | 97** | 4.00 | 760367-760447 | TAATGATAAATGTTGCATTTGATGCCTATATAAATTGAATCCAGTTATAAGTACGACGATAGCAATATTAATTAATATATA |
| 41 | 14** | 3.96 | 99370-99450 | TATAATTGGATATAACAAATAAATAATAATTATTGCAAAACACACCCGAAATTAATTATTATAAAAGTATATTCATAAAAG |
| 42 | 118** | 3.95 | 874250-874330 | TAGAGTTGTACTTTAGAGTTATTAAAAATAATAAAAAGGGTTTAAGTTGTTTATATTAAGGTATAAGTAAGTTATAATTAA |
| 43 | 46** | 3.94 | 362667-362747 | GTGACACCTCGTAATGATTTACTAAATGTTAATGTATCAGCAGAAATAAATATAAATTATCGTTTATCTGAATTAGGTTTT |
| 44 | 114*** | 3.93 | 862064-862144 | TGTCTTTTGTATAACGCTTTTAAAATACAACTACATAACGCTTTAGAATAATAATTGAATAAGTTTTTGTAATATCATCAG |
| 45 | 15*** | 3.82 | 100273-100353 | ATATTGACATTAAATAAATTGACAAAATAAGTAATTATTGTGAATCTAAAGTGAAATTTTTATAAAAAAATGTAATGATTC |
| 46 | 79*** | 3.82 | 685961-686041 | AACGCTATTGATGCCAATATCATTTATAATAAGTGATAAGCAAACTTTAAATTAATATCTTATATGAGGTTAACAATGAAT |
| 47 | 171** | 3.80 | 1235884-1235964 | ATATAAAAAAATTACGATTAACAAATATTAAAGCTTTTCAACATGATGCGACAAAACCTTATGATAAAACATACGATAAGA |
| 48 | 109*** | 3.77 | 844994-845074 | GTCCCCGAAATAATAAATCAAACAAATCATACTTTTGTAATTTACAAAAGCACATATAACTATATCAAATATAGCCCGATT |
| 49 | 348*** | 3.75 | 2494948-2495028 | TGTTTGGTTTTGGTCATTGTTTGTTGCATTTGAATTAGATTGTTGCTGGTTATCGTTTGCACTATTATCTTTATTATCTT |
| 50 | 86*** | 3.70 | 707750-707830 | GTAATGTATTAGGTAACCATTAAATTCGTTGTTATACGATTCTGATACAAGATTATGATAAAATAGCTTTAATTAAGAATT |
| 51 | 277*** | 3.70 | 2034076-2034156 | ATTGAGTAATTTCCAATCACATTCTTTTTTCTTATGATAGCTTAATTCATCACGTTGATAACTTATTAACGTTTCAACTTG |
| 52 | 167*** | 3.69 | 1223957-1224037 | ATAAGTCAAAACATTAATACCTATGATAAGTATCATTTATTAACATATGTATCATATTTTTAATCTTGCGTAATTTTTATC |
| 53 | 388*** | 3.68 | 2831332-2831412 | GATATTTAAAGTTGGACAATATTATATCTTGTGCAAAAATATAAATAAGTTATACATAATGTTAGAGAATCATGATATAAT |
| 54 | 83*** | 3.67 | 693985-694065 | GTTATTTATTTTAGATTGGAAAATGACATTATTAACATTTATAACGATACCGATATTCGTTTTAATTATGATTCCTCTAGGT |
| 55 | 216** | 3.62 | 1580164-1580244 | CATAACTATATAAATTTTGATAATTACGTTATACTTATCATTAATAAGTATCACATTAAACATGATACATGAATCGATATT |
| 56 | 80*** | 3.60 | 687933-688013 | GGGTGTTTTGACACAAAATGTTAATCATCAATGATAACAATGATATTTAAAAACTAAACTTATTTCAACTTACATGATTGT |
| 57 | 384*** | 3.59 | 2794459-2794539 | TATAAATATACGTTCTATGCCAATATCGAGTTTATTAAAATGATAAGCAATAAAGAATGAAAACATATTGATTACTTTTTC |
| 58 | 339*** | 3.56 | 2465045-2465125 | AATCTTATCAATGTTAGATAGGCCAATCATTAAGGCAGCAATAATAATCATAAAAATTACAAATGATTTAGCTTTAATTTT |
| 59 | 238** | 3.49 | 1771141-1771221 | TCCATTATAATAATTAAAGGTATATCTTTAGATAGTATGGTTTAATGTATCTTTATAAATATTATTTTATTATCGTTTATC |
| 60 | 307** | 3.42 | 2276676-2276756 | TAGGGTGACTTAATGGACAAAGTTAATAAGATCGCCAGAAATTGAATATAAAAAATATTAATATGGAAAGTACAGTGTGAG |
| 61 | 255*** | 3.41 | 1868915-1868995 | AGTATTTTTTTGAACGAGACATGCTAGACCTCCTGGATGATTATCATTTACATATCGTATCATAAACAATCATAAAACAAT |
| 62 | 287** | 3.41 | 2088202-2088282 | ATTATGCTGTTTTACTTTTTGAATGATAAGTAATTTTATGTTAAAAGTCTCCAGTTTGGATACAAAACGGTCGATAACATA |
| 63 | 387** | 3.40 | 2825666-2825746 | TAAATATTAATTGTATAAATCCTTATCTGAATTTTTCGAATCAAGAACGCAAATTATAAGCAACCACGTCATATATAGTTT |
| 64 | 72*** | 3.36 | 664167-664247 | TTTTAGTTCTCCTAATTGCAATAACTTTAGTTGATTAAGAATGATAAATATACCAAACAATGCAATGATAAATGTGATTGC |
| 65 | 176** | 3.33 | 1289286-1289366 | TATATTGTTAAAACAAATTTATCTTTAATTATACTATGTCTTCAGTCATTTTTCATCGAATAATCATTATATCAAAAATAA |
| 66 | 335*** | 3.28 | 2447168-2447248 | ATCGAAACATCAACACAACAATAAAATGATAAAAGGAAGTTATGATAGGCTCAAGGCCATTAAATCATAACTTCCTTTTTT |
| 67 | 152** | 3.26 | 1091861-1091941 | CATCAACTGTCCGACTATTCTTCATCAAAAAACCTGATAAAACAAATTGCCTTATCAGATTAGTATCATTTGTATAAGCAT |
| 68 | 246*** | 3.26 | 1831894-1831974 | CATACAGATGTCATGGGTGACGTGACAACTAACATTGGTGTTATTACATATGATAATGAAAACGCAGGTCTTTTCGGTATC |
| 69 | 122** | 3.24 | 892702-892782 | ATTATTTTTTGGTTTTGAATGATAATAAAACATTATCAATAAAACTTGCCATACGACGTAAATAATAAAACTAATTAATTG |
| 70 | 108** | 3.23 | 841179-841259 | AAAAAGCCCGACATTTACATCGCCGGACCTTACATACCAAGATAACTTATGCAAATCATAAGTACAAAAAATAATATTGAT |
| 71 | 21** | 3.21 | 136497-136577 | ACATTGTAATAGGTTTTTCAATTTATATGGTGGATGTTCTTGCAGTCCGTGTAGGATTAATCATAATGGTTATCATACAAA |
| 72 | 107** | 3.20 | 830824-830904 | AAAAAAAAGTTATTGACATATTATTGTCTAAATAATGTATAACCTTTGAACAACAATAATGATTATTAAATAGTAACTAAA |
| 73 | 95** | 3.20 | 746660-746740 | TTTGAAATACTTACATGTTGCTTACTAGTTGAATTATCATTGGAATACACATAATTATATCCATGATTTGTCTTAGAAATT |
| 74 | 198** | 3.15 | 1427708-1427788 | ACATCGCTGAAAGTGTTTATTTTTATTTAACAGGTACACATTACGAACAATAACTTAAAGTTATTACTATAAAATCCCTTA |
| 75 | 123** | 3.15 | 926139-926219 | AATATTATAAAGCGGTAATATGGAATCAAATAAATAAGCATAAGATTCATCACGATATAAAATAGCAATATCTTGATATCG |
| 76 | 273** | 3.13 | 2021447-2021527 | AATTCCCTTTTATAAGCTTGTAATCCTTGTTCTATTTGATAAGAAAAAGTTATATCATCAACAATCTCTTTCATACTATTT |
| 77 | 373*** | 3.12 | 2688615-2688695 | TTCGACATTTATCTCGTAAAGAAAAGTTACAACAATTGGTTGATAAGCAATGGTTATCAGAAGAACAATTCGACATTTTAC |
| 78 | 207** | 3.09 | 1534722-1534802 | ATGCATTACCAAGTATTATTACAAAAGAACAATTAAAAATGTTTGTTTATGATTATGATACGCATCTCATTAAAAATGTAA |
| 79 | 209** | 3.08 | 1548085-1548165 | GGTTATTATGAGAATTACAAAAGCTACATAAATTACTGTTAGTTTAAATTGAAATTTAAAAATGATAAGTAATCTTAATGC |
| 80 | 58** | 3.06 | 502375-502455 | TAAATAATGTTTGTAACTTATAGTTACGTTTTTTGGAATTAACGTTGACATATTGTCATTCAGTTTTCAATGTTCATTTTT |
| 81 | 172** | 3.05 | 1263264-1263344 | ACAAAAACATGGCAATCAAACCTTATCAACAATCATTATCAACGTTGAAACAACATGATTACCATAGTAACTATGTAAACC |
| 82 | 317** | 3.04 | 2340612-2340692 | ATGTCTTGGAATTAATTATTTAATAAGTTGTATAGCAGGTGGCGATATGAGATATATACACTTTTCTATTATTACATTAAT |
| 83 | 56** | 3.01 | 482758-482838 | CTGTACAATTATGTTATCATATATGAGTAGTTATAGCGCAAAACGTTAGCAATTCAGCGCACCCAACTTTTCATATAAACA |
| 84 | 229*** | 3.01 | 1669266-1669346 | ATTATAAAATAAATATAATATGTGATCAGCTATACTTATAGTATTTGTAAGATCGCGTAATTGCTTCATTGAAAAAATATA |
| 85 | 368** | 2.99 | 2653723-2653803 | ATATAATGAAATAATGATACTTCGTGTTACTATAACTGGATTTGCTATATTTACGTTATTGTTTGGATGGTTAAACGAATC |
| 86 | 355** | 2.95 | 2536519-2536599 | TCTATGATAAGGATAAAATTATCATTTTAGCTTATAAACCTTTGAGTAATGATGATGAAGTGCATTATTATGCATATGATT |
| 87 | 254** | 2.94 | 1865044-1865124 | AGAATAAAATATTTAGAAAAATCATTCATTTGCGTAATCGCTAAATTAAGTGTTAAATATAAGACATAAGTAATTAATTTA |
| 88 | 314** | 2.91 | 2321696-2321776 | GTATAGATTGTATTTAATAAGTTAATGTAATCCTTGAGCTCACGATTAATAAAATTCTATAACCTTAATTATTTTCTCGAT |
| 89 | 158*** | 2.89 | 1169382-1169462 | TTCATAAATTTGATATAAAACGCCTGATTCAGTATGGTTATCACTCACAATAATCCATTTAGACATTTTTATCACCTTCAA |
| 90 | 60** | 2.85 | 527557-527637 | ATGGTCAAATCGTGATGTTTCAACAAACTTTTCAATTGCGCGTGCTGCGTGTTCGATAACTTTTTCCATCGTTACCATTTC |
| 91 | 35** | 2.83 | 251405-251485 | TAGCACCTCAGTGAATTATAATTATGTTTATTTTATCATAATAGTGCATGTAAAATTCATGAATAAATTATTAAAAAAACA |
| 92 | 347** | 2.75 | 2492470-2492550 | AATCTCACTGTTCTGTAAAATGTTATCGATAACAAAATGCTATAGCCAGAGCATGATTTTAATGAAGCAATTACACTACTA |
| 93 | 102** | 2.75 | 801083-801163 | TTAATGTTGTTACAATAATCATTTTATTTGGTGTCGCGTTAGTGAGTCCTATTTACACACTATCGGAAGTTATCATTTTAA |
| 94 | 377*** | 2.72 | 2731257-2731337 | ATACTTTATAAATCGTAACTATTACGATTTATAATCAAAAACAATAACTTGAAATAGATCATTGAGGGAGTGTTAATATGC |
| 95 | 34*** | 2.71 | 250110-250190 | TGAATCCGTTAATGTAATAGCTGTAAAGTATAACGAAATGTTATTAAAGTCTTTTGTGAAATACGGTGTTAATCTATAAAA |
| 96 | 213** | 2.70 | 1576902-1576982 | CAAGGCGAAATTACTAAATACTGATGCGTGAGGACAACGCTTTAATTTCTATCATAAGTGAGTAATCATAATCATAAATTA |
| 97 | 208** | 2.68 | 1544477-1544557 | TCTGACATTTAAAATGAGATGTCATCCATTTTCTTAATTGAGCTTGAAAACAAACATTTATGAATGCACAATGAATATGAT |
| 98 | 74** | 2.67 | 666140-666220 | AATCATTTCTAAAACAACAGTCATATTACAATTATATTACAAATAATAATTACTACTTTTATATTTCACTTATCACTAAAA |
| 99 | 376** | 2.62 | 2729182-2729262 | TGTTGCTACAAATGTGGCAAAATATACAAATGTTTATCGTTCAAGCGCTACCTATTTTTATCACAATCTGTCTTATTGTTA |
| 100 | 81** | 2.57 | 689303-689383 | TAATAATTATCGATAATAATCATCTGCGCGCTTTTCAAAGCCTTAATTTGTTTTACCAATGTTCGATTAGTCATTTCTATC |
| 101 | 374** | 2.53 | 2703017-2703097 | ATAGTGGTAAAGATAATACTGCTGATATGATAAGCTTAATCAATTTATGTTGTAGCGCTTCAGCTTTGCGTGACGTTTGAT |
| 102 | 329** | 2.51 | 2419961-2420041 | GATTGATTATTATTTGAGTAGTTGTTGTAACGGCTGTAGTTATTGTAGCTATAACCGTTGTTGTAATTGTTATAGTTATTG |
| 103 | 336** | 2.50 | 2449386-2449466 | TTATGAAAAATATTATACGACTCCTGATTAATTTATGATTGACGTACTAAGGATTATCTTGTAACGTAAACGTAAATAAGC |
| 104 | 338** | 2.47 | 2462226-2462306 | GGCGGAATTTTGAATAGGTTATCAAGAATCGTATCAAATGAAGTGACATCATTAATATACAGTTTGAAATAAAGAAAATAT |
| 105 | 12** | 2.45 | 77607-77687 | GCAAACGATTATTACCACCAAACTATAAGTATTTAATTCCTTTTACTGCATTAGCTGGAGCAATCCTAATGATCATTTCAG |
| 106 | 30** | 2.44 | 233675-233755 | TTAATCCTATAAAAAGCAGGCGTTAAATGTAACAAGAGTTGCTGTGATAATTTCTCCTTATCTTCAATGTTAATAAAAGTG |
| 107 | 203** | 2.24 | 1467097-1467177 | GTACATAATACATAATAAGGAAGTGATACAATGGATGATAAGCAACACACATCTTCATCCGATGATGAACGCGCTGAAATT |

### Note: This table is sorted by the enrichment fold, which is derived from the data of BioSample accession numbers SAMN24812582. **, the peaks were identified from two out of three independent experiments; ***, the peaks are identified from all three independent experiments; ^#^, binding sites of CcpE by MEME analysis were underlined.
