# Supplementary material for: Citrate serves as a signal molecule to modulate carbon metabolism and iron homeostasis in Staphylococcus aureus
Source: PLoS Pathog. 2024 Jul 30;20(7):e1012425. doi: 10.1371/journal.ppat.1012425 (PMC11315280; doi:10.1371/journal.ppat.1012425)
Supplement: S5 Table — (DOCX) [file ppat.1012425.s013.docx]

**S5 Table. Plasmids and strains used in this study**

| Plasmids or Strains | Characteristics | Source |
| --- | --- | --- |
| pYJ335 | *E. coli*-*S. aureus* shuttle vector, Amp^r^ in *E. coli* and Cm^r^, Erm^r^ in *S. aureus* | [1] |
| p-*citB* | pYJ335 derivative carrying *citB* gene of *S. aureus* Newman in the downstream of  the *xyl*/*tetO* promoter | This study |
| p-*citZ* | pYJ335 derivative carrying *citZ* gene of *S. aureus* Newman in the downstream of  the *xyl*/*tetO* promoter | This study |
| p-*ccpE* | pYJ335 derivative carrying *ccpE* gene of *S. aureus* Newman in the downstream of  the *xyl*/*tetO* promoter | [2] |
| p-*pycA* | pYJ335 derivative carrying *pycA* gene of *S. aureus* Newman in the downstream of  the *xyl*/*tetO* promoter | This study |
| p-*fur* | pYJ335 derivative carrying *fur* gene of *S. aureus* Newman in the downstream of  the *xyl*/*tetO* promoter | This study |
| pKOR1 | Gene replacement vector for *S. aureu*s genes, Amp^r^ in *E. coli* and Cm^r^ in *S. aureus* | [2,3] |
| pKOR1:: ∆*citZ* | pKOR1 derivative, for deletion of *citZ* gene | This study |
| pKOR1:: ∆*sbnG* | pKOR1 derivative, for deletion of *sbnG* gene | This study |
| pKOR1:: ∆*ccpE* | pKOR1 derivative, for deletion of *ccpE* gene | This study |
| pKOR1:: ∆*pycA* | pKOR1 derivative, for deletion of *pycA* gene | This study |
| pKOR1:: ∆*isdC* | pKOR1 derivative, for deletion of *isdC* gene | This study |
| pKOR1:: ∆*fur* | pKOR1 derivative, for deletion of *fur* gene | This study |
| pCL-*lacZ* | *E. coli*-*S. aureus* shuttle cloning vector, single-copy integration vector in *S. aureus* | Laboratory stock |
| pCL-*pycA*-*lacZ* | pCL-lacZ derivative carrying *pycA* promoter | This study |
| pCL-*pycA*(G-N_11_-G)-*lacZ* | pCL-*pycA*-*lacZ* carrying guanine substitution mutant at the site of -287 bp and -275 bp from the translational start site of *pycA* | This study |
| pCL-*citB*-*lacZ* | pCL-lacZ derivative carrying *citB* promoter | [2] |
| pET28a | protein expression vector, Kan^r^ | Laboratory stock |
| pET28a::*ccpE* | pET28a derivative carrying *ccpE* of *S. aureus* Newman | [2] |
| pET28a::*sigma A* | pET28a derivative carrying *sigma A* of *S. aureus* Newman | This study |
| pET28a-*sumo* | pET28a derivative carrying a *sumo* tag | Laboratory stock |
| pET28a-*sumo*::*fur* | pET28a-*sumo* derivative carrying *fur* of *S. aureus* Newman | This study |
| pET28a-*sumo*::*isdC*_29-192_ | pET28a-*sumo* derivative comprising amino acid residues 29-192 of IsdC | This study |
| *E. coli* DH5a | For plasmid clone | Laboratory stock |
| *E. coli* BL21(DE3) | For protein expression | Laboratory stock |
| *S. aureus* RN4220 | Derivative of 8325-4 that accepts plasmids | Laboratory stock |
| *S. aureus* Newman | Wild-type, *S. aureus* ATCC 25904 | Laboratory stock |
| *S. aureus* Newman ^Tn^*citB* | *citB* transposon insertion mutant of Newman strain, with the transposon inserted 591 bp downstream the translational start site of *citB* | This study |
| *S. aureus* Newman ∆*citZ*^Tn^*citB* | *citZ* deletion and *citB* insertion mutant of Newman strain | This study |
| *S. aureus* Newman ∆*ccpE*^Tn^*citB* | *ccpE* deletion and *citB* insertion mutant of Newman strain | This study |
| *S. aureus* Newman ∆*ccpE* | *ccpE* deletion mutant of Newman strain, generated from pKOR1:: ∆*ccpE* | This study |
| *S. aureus* Newman/p | Newman strain carrying plasmid pYJ335 | [2] |
| *S. aureus* ^Tn^*citB/*p | ^Tn^*citB* strain carrying plasmid pYJ335 | This study |
| *S. aureus* ∆*citZ*^Tn^*citB/*p | ∆*citZ*^Tn^*citB* strain carrying plasmid pYJ335 | This study |
| *S. aureus* ∆*sbnG* ∆*citZ*^Tn^*citB/*p | ∆*sbnG* ∆*citZ*^Tn^*citB* strain carrying plasmid pYJ335 | This study |
| *S. aureus* ∆*ccpE*^Tn^*citB/*p | ∆*ccpE*^Tn^*citB* strain carrying plasmid pYJ335 | This study |
| *S. aureus* ∆*ccpE/*p | ∆*ccpE* strain carrying plasmid pYJ335 | This study |
| *S. aureus* ^Tn^*citB/*p-*citB* | ^Tn^*citB*strain carrying plasmid pYJ335::*citB* | This study |
| *S. aureus* ^Tn^*citB/*p-*pycA* | ^Tn^*citB*strain carrying plasmid pYJ335::*pycA* | This study |
| *S. aureus* ∆*ccpE/*p-*ccpE* | ∆*ccpE* strain carrying plasmid pYJ335::*ccpE* | This study |
| *S. aureus* ∆*citZ*^Tn^*citB/*p-*citZ* | ∆*citZ*^Tn^*citB* strain carrying plasmid pYJ335::*citZ* | This study |
| *S. aureus* ∆*sbnG* ∆*citZ*^Tn^*citB/*p-*sbnG* | ∆*sbnG* ∆*citZ*^Tn^*citB* strain carrying plasmid pYJ335::*sbnG* | This study |
| *S. aureus* ∆*ccpE*^Tn^*citB/*p-*ccpE* | ∆*ccpE*^Tn^*citB* strain carrying plasmid pYJ335::*ccpE* | This study |
| *S. aureus* Newman ∆*isdC* | *isdC* deletion mutant of Newman strain | This study |
| *S. aureus* Newman ^Tn^*srtB* | *srtB* transposon insertion mutant of Newman strain, with the transposon inserted 260 bp downstream the translational start site of *srtB* | This study |
| *S. aureus* Newman ∆*fur* | *fur* deletion mutant of Newman strain | This study |
| *S. aureus* ∆*fur*/p | ∆*fur* strain carrying plasmid pYJ335 | This study |
| *S. aureus* ∆*fur*/p-*fur* | ∆*fur* strain carrying plasmid pYJ335::*fur* | This study |
| *S. aureus* Newman ∆*pycA* | *pycA* deletion mutant of Newman strain | This study |
| *S. aureus* ∆*pycA*/p | ∆*pycA* strain carrying plasmid pYJ335 | This study |
| *S. aureus* ∆*pycA*/p-*pycA* | ∆*pycA* strain carrying plasmid pYJ335::*pycA* | This study |
| *S. aureus* Newman ∆*pycA*∆*ccpE* | *pycA* and *ccpE* double deletion mutant of Newman strain | This study |

Amp^r^, ampicillin resistance; Cm^r^, chloramphenicol resistance; Erm^r^, erythromycin resistance; Kan^r^, kanamycin resistance

**References**

1. Ji Y, Marra A, Rosenberg M, Woodnutt G (1999) Regulated Antisense RNA Eliminates Alpha-Toxin Virulence in *Staphylococcus aureus* Infection. Journal of Bacteriology 181: 6585-6590.
2. Ding Y, Liu X, Chen F, Di H, Xu B, Zhang X, et al. (2014) Metabolic sensor governing bacterial virulence in Staphylococcus aureus. Proceedings of the National Academy of Sciences 111: E4981-4990.
3. Bae T, Schneewind O (2006) Allelic replacement in Staphylococcus aureus with inducible counter-selection. Plasmid 55: 58-63
