# Supplementary material for: Citrate serves as a signal molecule to modulate carbon metabolism and iron homeostasis in Staphylococcus aureus
Source: PLoS Pathog. 2024 Jul 30;20(7):e1012425. doi: 10.1371/journal.ppat.1012425 (PMC11315280; doi:10.1371/journal.ppat.1012425)
Supplement: S6 Table — (DOCX) [file ppat.1012425.s014.docx]

**S6 Table. Primers used in this study**

| Primers | Sequence (5’ to 3’) | Purpose |
| --- | --- | --- |
| *citB-*F | CGTTTTCTAAGCATGATGAGTT | for p-*citB* |
| *citB-*R | GTGCCTCAAATACCGATTAAAA |  |
| *citZ*-F | AGTTGAAAGCCATTTCATAAAGA | for p-*citZ* |
| *citZ*-R | TATTGATTATTTTCTTTCTTCAAGC |  |
| *sbnG*-F | CAAATCCATTAGGTGTAGGAGG | for p-*sbnG* |
| *sbnG*-R | TTAGCCTTCATCCCCTTTCT |  |
| *pycA*-F | TTAGTTAGAAAATTCTAATAGTTTGAGG | for p-*pycA* |
| *pycA*-R | AATCATTTTAGTCAGTTGCTTTTTC |  |
| *fur*-F | ATTTTAATTATTAGTAGGGGAGTGG | for p-*fur* |
| *fur*-R | CGCAATTTACTATCCTTTACCTTT |  |
| *citZ*-up-F | GGGGACAAGTTTGTACAAAAAAGCAGGCTTTTTAACTGGATCTTTTGTTTCCCC | for pKOR1:: ∆*citZ* |
| *citZ*-up-R | TTACATCTTCATTTTTAATTTGTATTGAGATAAATTTCCCCTTTACTGTTTCT |  |
| *citZ*-down-F | TCAATACAAATTAAAAATGAAGATGTAA |  |
| *citZ*-down-R | GGGGACCACTTTGTACAAGAAAGCTGGGTGCATCTGAAATATAGTCACCATTCA |  |
| *sbnG*-up-F | GGGGACAAGTTTGTACAAAAAAGCAGGCTGTACACCCATGGCAGTTTGA | for pKOR1:: ∆*sbnG* |
| *sbnG*-up-R | GCATTCACAACTTCAAAAATATGTTTCTATGTTTCAGAGATAGTTGTTGC |  |
| *sbnG*-down-F | AACATATTTTTGAAGTTGTGAATGC |  |
| *sbnG*-down-R | GGGGACCACTTTGTACAAGAAAGCTGGGTGCGTACCTCCTCTTAATATGGCA |  |
| *ccpE*-up-F | GGGGACAAGTTTGTACAAAAAAGCAGGCTGATTTTGAAAACGATAAGAGTGTG | for pKOR1:: ∆*ccpE* |
| *ccpE*-up-R | TCATTATCAATTTCTACTTTTTCAAAGTCTTCAATCTTCATAATTTCCCC |  |
| *ccpE*-down-F | TTTGAAAAAGTAGAAATTGATAATGA |  |
| *ccpE*-down-R | GGGGACCACTTTGTACAAGAAAGCTGGGTTAAAATAAATGCAATAAATGGTAATAA |  |
| *pycA*-up-F | GGGGACAAGTTTGTACAAAAAAGCAGGCTATGCCTAGAGTATTAAGTAAACCAA | for pKOR1:: ∆*pycA* |
| *pycA*-up-R | AATAAATCGCCTGTCGCTATCGATACACTCCTCAAACTATTAGAAT |  |
| *pycA*-down-F | ATAGCGACAGGCGATTTATT |  |
| *pycA*-down-R | GGGGACCACTTTGTACAAGAAAGCTGGGTGAGTAAACGTGGATTACATAATTATC |  |
| *isdC*-up-F | GGGGACAAGTTTGTACAAAAAAGCAGGCTTAACTTTGTTAGTTTGTTGTGATTTAT | for pKOR1:: ∆*isdC* |
| *isdC*-up-R | AATAATGCTAAGGATGCAACTGAACATAATCCTCCTTTTTATGATTG |  |
| *isdC*-down-F | CAGTTGCATCCTTAGCATTATT |  |
| *isdC*-down-R | GGGGACCACTTTGTACAAGAAAGCTGGGTACGACGCTTATCACTAAAATGG |  |
| *fur*-up-F | GGGGACAAGTTTGTACAAAAAAGCAGGCTAACCAAGCACGTCATGTGATGT | for pKOR1:: ∆*fur* |
| *fur*-up-R | GCGCCTTCTTTTCTTAAATCAAGATGTCCACTCCCCTACTAATAAT |  |
| *fur*-down-F | TTGATTTAAGAAAAGAAGGCGC |  |
| *fur*-down-R | GGGGACCACTTTGTACAAGAAAGCTGGGTAGCAACAGATTGCAAAATTGAA |  |
| *pycA*-*lacZ*-F (*Eco*RI) | CCG*GAATTC*AAAAAATACTAGCCAATATTTAGTACG | for pCL-*pycA*-*lacZ* |
| *pycA*-*lacZ*-R (*Kpn*I) | CGG*GGTACC*TAACTTTTTTATTTGTTTCAATAGCG |  |
| *pycA* (G-N_11_-G)-*lacZ*-F | TGAGAAGATAAAGTTGTCGCTATTGATAATAAATGTTGATG | for pCL-*pycA* (G-N_11_-G)-*lacZ* |
| *pycA* (G-N_11_-G)-*lacZ*-R | GCGACAACTTTATCTTCTCATCATGATTCTAATTTCGCCA |  |
| pET28a::*ccpE*-F (*Bam*HI) | GGG*GGATCC*ATGAAGATTGAAGACTATCGTTTACT | for pET28a::*ccpE* |
| pET28a::*ccpE*-R (*Xho*I) | CCG*CTCGAG*TCTAAAACTACGCCTTTGGTTGT |  |
| pET28a::*sigma A*-F (*Bam*HI) | GGG*GGATCC*ATGTCTGATAACACAGTTAAAATTAAA | for pET28a::*sigma A* |
| pET28a::*sigma A*-R (*Xho*I) | CCG*CTCGAG*TTAATCCATAAAGTCTTTCAAACGT |  |
| pET28a*-sumo*::*fur*-F (*Kpn*I) | CGG*GGTACC*TTGGAAGAACGATTAAATCGC | for pET28a*-sumo*::*fur* |
| pET28a*-sumo*::*fur*-R (*Hind*III) | CCC*AAGCTT*CTATCCTTTACCTTTAGCTTGGC |  |
| pET28a-*sumo*::*isdC*_29-192_-F (*Kpn*I) | CGG*GGTACC*GCAGATAGCGGTACTTTGAATT | for pET28a-*sumo*::*isdC*_29-192_ |
| pET28a-*sumo*::*isdC*_29-192_-R (*Hind*III) | CCC*AAGCTT*TGTTTGTGGATTTTCTACTTTG |  |
| *citB*-RT-F | CTTCGTCAAGAAGATGATTTTG | for RT-qPCR of *citB* |
| *citB*-RT-R | CGCCTTCATTTCCATCTTTTC |  |
| *isdC*-RT-F | TACAACGATTTTAGCGTTAATTATCATC | for RT-qPCR of *isdC* |
| *isdC*-RT-R | TTGACGTGTCATTGGTATTGTAT |  |
| *pxpB*-RT-F | GGACCAGATATTGAAGAAGTAGC | for RT-qPCR of *pxpB* |
| *pxpB*-RT-R | CCTGGCATAAATCCTAGCATATATA |  |
| *isdA*-RT-F | CAGTAAGTATCAATCAGAACAACG | for RT-qPCR of *isdA* |
| *isdA*-RT-R | TTCTGTTGCCGCATTGACTT |  |
| *sirA*-RT-F | ATGAATAAAGTAATTAAAATGCTTGTT | for RT-qPCR of *sirA* |
| *sirA*-RT-R | TTAATTGAAGTTGTTTCCTTATCTT |  |
| *sbnA*-RT-F | AAAGTCAAGCATGTCACGATT | for RT-qPCR of *sbnA* |
| *sbnA*-RT-R | CCAGGATTCATATACTCTAACTTTG |  |
| *pycA*-RT-F | GGTACAGACGGTCCAATTAAA | for RT-qPCR of *pycA* |
| *pycA*-RT-R | TTCTCATACCTTTACCGCCG |  |
| 16S-RT-F | GGCAAGCGTTATCCGGAATT | for RT-qPCR of 16S rRNA |
| 16S-RT-R | GTTTCCAATGACCCTCCACG |  |
| *pycA*-RT-trans-F | ACAAATAAAAAAGTTACTTGTTGCT | for RT-qPCR of *pycA in vitro* transcripts |
| *pycA*-RT-trans-R | ATCTGCTTTATATCTATGTAATGAACTT |  |
| *isdC*-EMSA-F | TAATTCAAAGTACCGCTATCTGC | for EMSA of *isdC* |
| *isdC*-EMSA-R | CGTTGTTCTGATTGATACTTACTGT |  |
| *citB*-EMSA-F | GGCTGGGACATAAATCAATATT | for EMSA of *citB* |
| *citB*-EMSA-R | AAATCATAGTAAGTATAACTTTGGCC |  |
| *murA-*EMSA-F | GGATAAAATAGTAATCAAAGGTGG | for the control DNA of EMSA of *citB* |
| *murA-*EMSA-R | TCGTTAATACGTTCAATGTCTGC |  |
| *citB*-EMSA-F2 | TCTGATTAAAAAGTCAAAACCTAAAT | for the control DNA of competition assay |
| *isdC*-footprinting-F | FAM-TAATTCAAAGTACCGCTATCTGC | for foot printing of *isdC* |
| *citB*-footprinting-F | FAM-GGCTGGGACATAAATCAATATT | for foot printing of *citB* |
| *pycA*-footprinting-F | FAM-CAAAATTTAACATTCTTGAGACAAT | for foot printing of *pycA* |
| *pycA*-footprinting-R | ATTGGACTGATGAGTTCAATAACG |  |
| *pxpB*-footprinting-F | FAM-GTAAAAATTTTCCATCAGACCG | for foot printing of *pxpB* |
| *pxpB*-footprinting-R | AATCATAATTGTCTGCTCGTTAAT |  |
| *citZ*-footprinting-F | FAM-TTTTCAGCCATAATACATCTCCC | for foot printing of *citZ* |
| *citZ*-footprinting-R | AATTCTGCCATGATAAATTTCCC |  |
| *pycA*-race-1 | GATTACGCCAAGCTTTCCGGCATGAAATCATTTAACGGATCTTCTGTTGTA | For 5’-RACE |
| *pycA*-race-2 | GATTACGCCAAGCTTTAGCGGGAAACCAGCTTCTTCTGCAAATTCTTTTG |  |
| M13F | TGTAAAACGACGGCCAGT |  |
| *isdC*-trans-F | GGGTGTTGCATATAGTCATCCA | for *in vitro* transcription of *isdC* |
| *isdC*-trans-R | CTTACTTCAAATTCAGAAGTGCG |  |
| *citB*-trans-F | ATTTCTGTCCCACTCCCATC | for *in vitro* transcription of *citB* |
| *citB*-trans-R | GTAATATCTCCCCCAACGTCA |  |
| *pycA*-trans-F | ATTGGACTGATGAGTTCAATAACG | for *in vitro* transcription of *pycA* |
| *pycA*-trans-R | ACGCGCAAATTGTTCATTTT |  |
