# Supplementary material for: Citrate serves as a signal molecule to modulate carbon metabolism and iron homeostasis in Staphylococcus aureus
Source: PLoS Pathog. 2024 Jul 30;20(7):e1012425. doi: 10.1371/journal.ppat.1012425 (PMC11315280; doi:10.1371/journal.ppat.1012425)
Supplement: Attachment — Submitted filename: point-to-point response letter.pdf [file ppat.1012425.s016.pdf]

**Point-to-point response letter**

Reviewer #1: In the resubmission, the authors have addressed some, but not all of my concerns. Notably, the manuscript remains very dense, and while the study is definitely in my area of expertise, I had a very difficult time getting through the experiments and understanding the model and conclusions. I don't think the crystallography adds much to the story, and is incremental. The regulatory model with CcpE and Fur is not clear.
Response: Thank you for your feedback. In response, we have removed the crystallography data and the corresponding statement in the revised manuscript. Additionally, we have removed the streptonigrin experiments in accordance with the comments from Reviewer #2. We have carefully condensed the manuscript further to enhance its overall clarity.

Reviewer #2: I want to thank the authors for addressing my initial concerns. The study is very valuable, novel and effectively showcases the impact of citrate on iron homeostasis and the metabolism of S. aureus. Upon reading the revised manuscript, I understand that the model presented indicates an increase in the intracellular pool of citrate has two major consequences for S. aureus. First, this activates CcpE through direct binding of citrate, leading to differential regulation of metabolism, particularly impacting certain nodes of central metabolism. Convincing evidence by the authors shows that citrate-activated CcpE competes with the Fur-binding site in promoter regions, leading to the activation of Fur-repressed genes. Separate from CcpE, the authors also demonstrate that citrate itself can decrease the repressor activity of Fur by depleting cells of divalent ions required for Fur function. Although the evidence for the latter consequence is strong, there are some aspects in this line of inquiry that could be further clarified.
Response: We are pleased to hear that you find our study valuable, novel, and effective in showcasing the impact of citrate on iron homeostasis and the metabolism of *S. aureus*.

In the revised manuscript, we have carefully condensed and revised the content to enhance clarity.

Reviewer #3: The authors present an extensively modified manuscript. My comments were addessed sufficiently.
Response: We are pleased to know that we have sufficiently addressed your comments.

Part II – Major Issues: Key Experiments Required for Acceptance
Please use this section to detail the key new experiments or modifications of existing experiments that should be absolutely required to validate study conclusions.

Generally, there should be no more than 3 such required experiments or major modifications for a "Major Revision" recommendation. If more than 3 experiments are necessary to validate the study conclusions, then you are encouraged to recommend

"Reject".

Reviewer #1: • Lines 286-291: The model is difficult to conceptualize. CcpE acts as an apparent repressor, and knocking out ccpE results in de-repression. But this regulation is far from the core promoter and transcription +1. How does it work? Also, mutating doesn't seem to tell you much about regulation by CcpE if the basal promoter activity is affected. Again, still unclear how this would work if it's so far upstream of the promoter.

Response: We apologize for any confusion caused. The binding site of CcpE is situated in relation to the start codon of *pycA*, as detailed in the legend accompanying Fig. 2.

In response to your feedback, we conducted a 5'-race experiment and included the results in the revised manuscript as follows (Line 258-261): "Through 5'-rapid amplification of cDNA ends (5'-RACE) experiments, we determined that the transcriptional start site of *pycA* is located 275 bp upstream of its start codon, specifically at the A site of the potential T-$N_{11}$-A motif".

• Lines 318-320: If CcpE binds to promoters and enhances expression of isdA, etc., then I would expect increased expression in citB mutant, and less in citB citZ mutant, and less in citB ccpE mutant. Doesn't appear to be the case with the values below the genes. (Fig S7)

Response: We sincerely thank you for your attentive reading.

We showed that CcpE binds to promoters and can enhance the expression of *isdA* in the $^{Tn}$*citB* mutant cultured in TSB for 12 hours. Similar results were observed for Fur-regulated genes such as *isdC*, *isdI*, *fhuD2*, and *sbnA* (Fig. S6). Thus, CcpE appear to enhance the expression of Fur-regulated genes under this testing conditions (iron-replete) in the $^{Tn}$*citB* mutant.

However, under the testing conditions, the $^{Tn}$*citB* mutant exhibited reduced expression of *isdA* and *isdC*, whereas it showed elevated expression of *isdI*, *fhuD2*, and *sbnA*, when compared to the wild-type Newman strain (Fig. S6).

A study (PMID: 38869061) that emerged during the second round of revisions for this manuscript, revealed that aconitase not only serves as its traditional enzymatic role but also acts as an RNA-binding protein to downregulate its own mRNA and the *citZ*-*citC* mRNA, while upregulating *pycA* mRNA under iron-deprived conditions. This study further demonstrated that IsrR, a small regulatory RNA that is repressed by Fur, downregulates CitB and CcpE through post-transcriptional control mechanisms. Thus, *S. aureus* appear to employ a sophisticated regulatory pathway involving Fur, IsrR, CcpE, and CitB to adapt to iron-limited environments (PMID: 38869061).

Under iron-limited conditions (i.e., TSB + 1 mM DIP), the $^{Tn}$*citB* mutant demonstrated elevated expression of Fur-regulated genes such as *sirA*, *sbnA*, *isdC*, and *isdA*, in contrast to the wild-type Newman strain (Fig. 5D and E). These findings support the hypothesis that CcpE can bind to promoters and enhance expression of Fur-regulated genes (Fig. S6). Nevertheless, the detail mechanism by which CitB

modulates these genes, particularly in the wild-type *S. aureus* strain cultured in iron-replete conditions (i.e., TSB medium), remains an unanswered question that necessitates further in-depth investigation.

In light of your valuable feedback, we have incorporated the new reference (PMID: 38869061) into the revised manuscript (line 339-346) as indicated: However, the relationship between iron homeostasis and citrate accumulation in *S. aureus* appears to be intricate and multifaceted. Fur not only binds to the promoter and triggers *citB* expression *in vitro* and *in vivo* (S8A-E Fig.), but it also represses IsrR, an sRNA that in turn downregulates CitB and CcpE through post-transcriptional control mechanisms [34]. Furthermore, the revelation that Fur serves an activator for *citB* aligns with earlier findings in *E. coli,* reinforcing the concept that Fur links iron transport and utilization enzymes with negative-feedback loop pairs for iron homeostasis [35].

- For CcpE binding to the isd promoters – looks like the binding is intrinsic and not dependent on citrate.

Response: In the DNase I footprinting experiment (Fig. 4C), the inclusion of citrate resulted in the emergence of hypersensitive sites, a pattern distinctly different from that observed when CcpE was present alone. This finding suggests that citrate modifies the DNA-binding properties of CcpE.

Our EMSA experiment also showed that citrate enhances the binding capacity of CcpE to the *isdC* promoter. The results of this experiment have been incorporated into the point-to-point response letter as follows (Fig. R1).

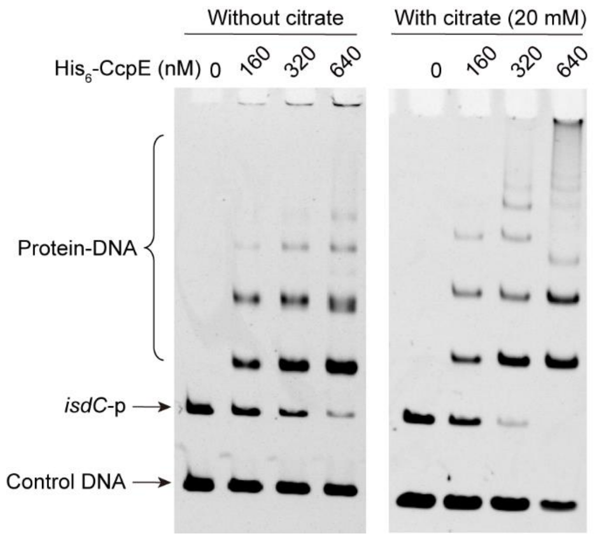


**Fig. R1 Citrate can enhance the binding capacity of CcpE to the *isd* promoter.** For the right panel, a final concentration of 20 mM sodium citrate was used in the binding buffer (10 mM Tris-borate, pH 8.0; 1 mM MgCl2; 25 mM KCl; 100 µM MnCl2; and 50 µg/mL BSA). A 200-bp DNA fragment covering nucleotide -127 to nucleotide +73 relative to the start codon of *citB* was used as the control DNA.

- Fig 5D – The figure legend lacks detail to help the reader to interpret the EMSAs. In

Fig S8, what is meant by P2 form of IsdC?

Response: In the revised manuscript, we have refined the figure legend as follows (line 1451-1453): EMSA experiments illustrating the impact of CcpE on the protein-DNA complexes formed by the Fur protein with the *isdC* promoter DNA (Fur-*isdC*).

Additionally, in response to your feedback, we have excised the right panel of Fig. S8 from the current manuscript version. The panel depicted the relative abundance of the P2 form of IsdC.

- Lines 333-345: Fig 5E – the western blot isn't convincing for showing the effect of citrate activating CcpE and activating isdC expression

Response: The aforementioned statement, "which supports the notion that citrate activates CcpE [12], which in turn activates *isdC*.", has been removed from the current version of the manuscript.

- Lines 355-357: I don't see evidence of this. CcpE appears to bind multiple Fur-regulated genes. RNA-Seq shows a positive effect of CcpE on isd gene expression

Response: The previous assertion, "Since CcpE appears to directly activate multiple Fur-regulated genes", has been revised as "Since CcpE is observed to bind to the promoter region and stimulates the expression of multiple Fur-regulated genes".

- Lines 362-373: With the DIP treatments, could it be that Fur-mediated repression is lifted? Wouldn't you have to knock out ccpE to show that CcpE is activating under these conditions? I don't think it's clear that the authors have iron deficiency augments the regulatory function of CcpE in S. aureus

Response: In light of your valuable feedback, we investigated the expression levels of *citB* in both wild-type Newman strain and the Δ*citZ*, Δ*citZ*Δ*sbnG*, and Δ*ccpE* mutants using qRT-PCR assays. The results from these experiments have now been integrated into the current version of the manuscripts (Fig. 4F and G). These results indicate that iron deficiency enhances the regulatory role of CcpE.

- Fig 6A and Lines 375-385: I'm confused here. If CcpE negatively regulates iron uptake and Fur negatively regulates uptake, then one would expect either no change or an increase in iron levels in the ccpE mutant, right? Why the change in the citB background? The changes are potentially statistically significant but are they biologically significant? Also, using Dunnett;s test compares samples to a control. This looks like a Tukey's test. Please verify. I guess having a model would be helpful here.

Response: We apologize for any confusion this may have caused. In response to your valuable feedback and that of Reviewer #2. We have excised the previous Fig. 6A-C from the current manuscript.

- Fig 6F is not very convincing – authors state on Lines 411-412 "…citrate has the ability to alleviate the inhibition of isdC transcription by the recombinant Fur protein." The effect is small.

Response: Under our experimental setup, the inclusion of citrate was observed to

mitigate the inhibitory influence of Fur on the *in vitro* transcripts of the *isdC* gene, leading to an approximately 2.6-fold enhancement in the levels of *isdC* mRNA.

- At the end of section, line 418: I'm lost…the manuscript is dense and the writing is not clear.

Response: The aforementioned statement, "both independently and in a manner dependent on CcpE.", has been removed from the current version of the manuscript.

Following your insightful comments and those of Reviewer #2, we have carefully condensed and revised the manuscript. We are confident that the presentation and interpretation of the findings has been significantly improved.

Reviewer #2: 1. While the authors have shown that CcpE differentially regulates various metabolic genes (Fig S6), it would be helpful to compare this to the metabolomics data provided and highlight its actual metabolic consequences to the cell. Also, a pathway enrichment analysis might also highlight some primary impacts of CcpE in S. aureus.

Response: In light of your insightful feedback, we have incorporated a new paragraph into the revised manuscript (line179-190) as follows: We conducted a differential networking analysis using Joint-Pathway Analysis [22] to further our investigation. This integrated analysis of transcriptomics and metabolomics facilitated a comprehensive visualization of the interactions between differentially expressed genes and perturbed metabolites. By integrating the data, we identified 11 and 8 enriched pathways (with $p$-value < 0.05 and an impact value greater than 1) in the Δ*citZ*$^{Tn}$*citB* (S3A Fig.) and Δ*ccpE*$^{Tn}$*citB* (S3B Fig.) mutants, respectively, as compared to the $^{Tn}$*citB* mutant (S3 Fig.). Notably, all of the enriched pathways identified in the Δ*ccpE*$^{Tn}$*citB* mutant were in complete concordance with those found in the Δ*citZ*$^{Tn}$*citB* (S3 Fig.). These pathways include pyruvate metabolism, purine metabolism, alanine, aspartate and glutamate metabolism, arginine biosynthesis, glycine, serine and threonine metabolism, pyrimidine metabolism, glycolysis or gluconeogenesis, and pentose phosphate pathway (S3 Fig.).

2. Line 112: The authors indicate that the intracellular pool of iron in the citB mutant was steady despite the citZ mutation and this surprising finding could not be attributed to sbnG. To me, this is confusing since there shouldn't be any alternate source of citrate, or at least none the authors have identified. I wonder if what the authors are detecting by mass spectrometry is isocitrate in the cell, formed through the glutamate/2-oxoglutarate node within the TCA cycle. Note that isocitrate is not readily distinguished from citrate by mass spectrometry. If the authors have not ruled out isocitrate, they should state this possibility in the manuscript and reassess how this impacts the rest of their story. For instance, does isocitrate impact CcpE or chelate iron?

Response: We appreciate your insightful observation. Indeed, citrate and isocitrate possess the same molecular weight, which can make them challenging to differentiate during LC-MS/MS analysis due to their very similar retention times. This similarity can be further complicated by issues such as peak tailing or broadening.

In contrast to citrate, isocitrate does not appear to affect the DNA-binding affinity or the thermal stability of protein CcpE, as indicated by the study with the PubMed identifier (PMID): 25368190. Thus, it seems that isocitrate does not exert a direct influence on the activity or function of CcpE.

3. The most difficult aspect to interpret in the manuscript is the impact that the citZ mutation has on intracellular iron. If the authors' interpretation that increased intracellular citrate in the citB mutant decreases the intracellular iron pool to impact Fur activity is true (Fig 6A), why does this mutant have the most sensitivity to Streptonigrin compared to the wild type? Even more confusing is the observation that the citZcitB double mutant is significantly less sensitive than WT to Streptonigrin, suggesting that iron levels are very low in the citZcitB mutant despite less citrate (Fig 6B). The authors need to measure the levels of intracellular iron in the WT and citZ mutants if readers are supposed to make sense of the Streptonigrin experiments.
Response: We apologize for any confusion this may have caused.

In response to your perceptive feedback and the observations of Reviewer #1, who noted that the previous version of the manuscript was quite dense, we have undertaken a comprehensive revision. To improve the readability and streamline the content, we have removed the former Fig. 6A-C, along with the former Fig. 2, which contained the crystallography data and its associated information.

Reviewer #3: (No Response)

Part III – Minor Issues: Editorial and Data Presentation Modifications
Please use this section for editorial suggestions as well as relatively minor modifications of existing data that would enhance clarity.

Reviewer #1: • Line 70: Use of a review on Salmonella is odd, since there are several now that focus on S. aureus. Strongly suggest citing Richardson AR 2019 (DOI: 10.1128/microbiolspec.GPP3-0011-2018)
Response: We have incorporated the suggested reference appropriately (ref8). We have excised the reference pertaining to the *Salmonella* review from the revised manuscript.

• Line 83: please rephrase: "senses and responds to citrate, an intermediate of the TCA cycle…"
Response: We have revised the sentence in accordance with your recommendation.

• Line 104: elevating: change to elevates
Response: We have changed the term "elevating" to "elevates".

• Fig S1: It would help to have the y-axes at the same scale to make comparisons easier.
Response: In the revised manuscript, we have standardized the y-axes of Fig. S1 to maintain a consistent scale.

• Fig 1D: I still find the Venn diagrams difficult to interpret. Perhaps a better figure legend describing the comparisons and what is meant by citrate-regulated and CcpE-regulated would help…or setting up the comparisons in the manuscript body text

Response: In the revised manuscript, we have detailed the comparisons and clarified the meanings of "citrate-regulated" and "CcpE-regulated" within the figure legend of Fig. 1.

• Line 159: a round – change to around or better, approximately

Response: We have updated the terminology, replacing "a round" with "around".

• Lines 179-218: Still don't know the structure-function studies add anything – seems more of a detail. And, it seems to interrupt the flow – gene expression, then structure, then binding site identification for direct targets.

Response: We have excised the previous content from the revised manuscript, which included the crystallography data and its corresponding information.

• Line 234 and Fig S5A…check fold-enrichment values – they don't match

Response: In the main text, we reported average fold-enrichment values; however, in the accompanying figure featuring IGV views displays the fold-enrichment data derived from a single replicate. We have meticulously verified that this discrepancy does not affect the accuracy of our statement.

• Fig 4AB, no citrate added? Label as such, why not? Stronger effect but did not test?

Response: We apologize for any confusion this may have caused. In the revised manuscript, we have amended the Y-axis label to read "Relative expression of *pycA*", replacing the previous label of" Relative expression of *pycA in vivo*".

• Lines 295-314: These experiments are designed to test the hypothesis that repression by CcpE (when activated by high intracellular citrate) will generate phenotypic auxotrophy. The data are consistent, but the writing could be refined to make this clearer.

Response: By following your comments, we have refined this paragraph. Please see line 269-287 for more detail.

• Lines 311-314: Not clear on the logic: what the DIP treatment is doing here. Does it somehow increase citrate levels, leading to increased repression in a CitZ, SbnG, and CcpE-dependent manner? Please clarify.

Response: We apologize for any confusion this may have caused. We have deleted the sentences in the revised manuscript.

In response to your insightful feedback, we performed qRT-PCR assays to investigate the impact of DIP treatment on the expression levels of *pycA*, *citB*, *pxpB*, and *isdC* in both the wild-type Newman and the isogenic Δ*citZ*, Δ*citZ*Δ*sbnG*, and Δ*ccpE* mutant strains. The findings have been incorporated into the revised

manuscript under a sub-section entitled "Citrate accumulation and activation of CcpE are triggered by iron starvation". The pertinent data are now included in lines 335-338 and are illustrated in Figures 4F and 4G. The outcomes suggest that the changes in gene expression prompted by DIP treatment are largely dependent on the activity of CcpE.

- Lines 324-326: in vitro transcription Fig 5B – is this citrate dependent CcpE enhancement? Also stats should be ANOVA with Dunnetts test

Response: In the Figure, we showed the effect of CcpE on the *in vitro* transcription of *isdC* in the presence of citrate. The statistical analysis was re-performed, and One-way ANOVA with Dunnett's multiple comparisons test was used now (Fig. 4B).

- Fig S8B legend: is this the quantitative analysis of the western in Fig 5E? Please clarify in legend

Response: Yes, the figure presents a quantitative analysis corresponding to the western blot depicted in Fig. 4E (previous Fig. 5E). We have revised the figure legend accordingly in the updated manuscript to reflect this detail.

- Lines 346-351: They don't seem to contribute to the story and just make things less clear.

Response: We have excised the mentioned paragraph from the current manuscript.

- Lines 371-373: Fur bound to iron typically acts as a repressor, but your data show a stimulatory role. This seems consistent with a report in E. coli for AcnA (aconitase). Please mention this. https://www.ncbi.nlm.nih.gov/pmc/articles/PMC4167408/

Response: We referenced this matter in the revised edition of the manuscript. For further details, please refer to Line344.

- Regarding the above point, please add some background information on Fur and the mechanism of regulation.

Response: We have incorporated additional background information regarding Fur and its regulatory mechanism into the revised manuscript. Please refer to lines 339-346 for the details.

- Line 437-438: Instead of "…independent of citB" do you mean in the citB mutant?

Response: Yes, we have revised the sentence to read, "…to regulate the pathogenicity of *S. aureus* in the $^{Tn}$*citB* mutant."

Reviewer #2: 1. Line 126: Change 'incubation' to 'growth of S. aureus strains'.
Response: We have polished this description in accordance with your suggestion.

2. Line 128: Describe the strains used here.
Response: We have detailed the strains in accordance with your suggestion.

3. Line 288: Remove the word ‘was’.
Response: Thank you for your thorough review. We have removed it.

4. Line 522-524: When oxygen is present, S. aureus does not thought to actively metabolize glucose through the TCA cycle due to CcpA repression. Thus, acetate is the primary byproduct of glucose metabolism.
Response: We have revised the sentences in accordance with your recommendations. Please refer to lines 469-473 for the details.

5. Line 482: Correct ‘post-experimental’ to ‘post-transcriptional’.
Response: We have corrected it.

Reviewer #3: (No Response)
Response: Thank you!
