# Supplementary material for: Citrate serves as a signal molecule to modulate carbon metabolism and iron homeostasis in Staphylococcus aureus
Source: PLoS Pathog. 2024 Jul 30;20(7):e1012425. doi: 10.1371/journal.ppat.1012425 (PMC11315280; doi:10.1371/journal.ppat.1012425)
Supplement: Attachment — Submitted filename: Point-to-point response letter-Lan.docx [file ppat.1012425.s015.docx]

**Point-to-point response letter**

Reviewer's Responses to Questions

Part I - Summary

Please use this section to discuss strengths/weaknesses of study, novelty/significance, general execution and scholarship.

Reviewer #1: In the manuscript “Citrate serves as a signal molecule to modulate carbon metabolism and iron homeostasis in Staphylococcus aureus”, Chen et al. extend a previous study on the CcpE regulatory protein that was shown to bind citrate and allosterically regulate its DNA-binding activity, resulting in the differential expression of dozens of genes. CcpE is a major positive regulator of TCA-cycle activity, but also regulates the expression of staphylococcal virulence genes. A growing body of evidence demonstrates the linked nature of physiology and virulence; thus, a better understanding of the molecular mechanisms underpinning these linkages is significant and may lead to new therapies to combat this potentially devastating human pathogen.

In the current study, the authors use a citB mutant (blocked for the TCA cycle and accumulates citrate) and a citB citZ mutant (blocked for the TCA cycle, does not accumulate citrate) to interrogate the transcriptome, metabolome, and virulence phenotypes. This is a clever approach, as the comparison avoids confounding effects of disrupting the TCA cycle generally on cell physiology. In doing so, they identify differentially regulated genes dependent and independent of citrate levels, and metabolite changes dependent and independent of ccpE. This is definitely a resource for others in the field. They solve a crystal structure of CcpE’s regulatory domain with citrate, and identify additional amino acid residues that indirectly affect the binding of citrate. They also perform IDAP-Seq, a genome-wide DNA-binding assay to identify direct targets of CcpE. The authors identify a CcpE DNA-binding motif that is strikingly similar to the Fur binding motif. Admittedly the motif is highly degenerate (TWWDWWWWRVTTATCATT). They identify and they identify a subset of genes that are regulated by both Fur and CcpE, and use EMSA, footprinting, and in vitro transcription in an attempt to demonstrate direct binding and regulation. There is limited testing of the role of bases in the motif in transcription factor binding. In the end, the authors come up with a model whereby CcpE modulates iron homeostasis, connecting CcpE and Fur activities.

In the end, the manuscript is very long and dense, and it is very difficult to assimilate all the information. It seems as though there could be multiple, independent manuscripts here. Importantly, I was puzzled why the authors did not bring up the previously described second citrate synthase SbnG (https://pubmed.ncbi.nlm.nih.gov/24666349/). This seems like an important consideration, or at least one to mitigate early in the manuscript text if not important. Activity of a second citrate synthase might affect intracellular pools, at least under iron limitation. The structural studies included here seem incremental, and it is unclear what they really add to our knowledge of CcpE function, and the overall story herein. Generally, there are so many things being examined in the manuscript that the focus is lost, and the reader is left trying to understand the overall model. For instance, do CcpE and Fur compete for binding of target genes? Which motif(s) are required for these regulations? In many cases, there are missed opportunities to test hypotheses directly; instead, the authors stick to the same series of mutant strains and complement strains, and show correlations. In some places the data may be over-interpreted. At best, the results of those experiments suggest, not indicate, molecular mechanism. Overall, the writing could be made clearer, and there are a number of misspellings. The authors frequently use the phrase “To further investigate X, we did Y” – this is vague, and it would be more helpful to use more intentional language and clear about what they are testing. Finally, there is frequent use of t-tests to test statistical significance where one-way ANOVA and post test should be used, to avoid errors with multiple hypothesis testing.

Response: We are delighted to hear that you find our study to be a valuable resource for others in the field.

We are also thankful to you for offering constructive criticism on our study. In response to your feedback, we have carefully revised and condensed the manuscript to enhance clarity in its presentation. Additionally, we have included a citation to the SbnG paper (*Mol Microbiol*. 2014, 92:824-39, Ref 41 in the revised manuscript). Furthermore, we conducted additional EMSA experiments, which revealed that CcpE and Fur compete for binding to the intergenic region of *isdA* and *isdC* (Fig. 5D and S8A). Moreover, the ANOVA test was employed instead of *t*-tests for comparing multiple groups in the revised manuscript (in Fig. 1B, 2D, 4A, 4B, 4D, 6A, 6G, 6H, S1A and S1B).

Reviewer #2: In their manuscript, Chen et al. investigate the influence of citrate on the regulation of carbon and iron metabolism in S. aureus through CcpE, a LysR-type transcriptional regulator. They distinguish between direct and indirect CcpE targets using metabolomics, transcriptomics, and IDAP sequencing and determine the crystal structure of CcpE bound to citrate. The manuscript also discusses CcpE's negative regulation of pycA and its positive regulation of Fur-regulated genes. Citrate's role in iron homeostasis and its impact on Fur regulatory activity are emphasized. Additionally, the authors demonstrate citrate's effect on S. aureus pathogenesis through mouse and G. mellonella infection models. Overall, the study successfully highlights a very underappreciated role for citrate in modulating gene regulation in S. aureus.

Response: We are pleased to receive your acknowledgment of our study highlighting a very underappreciated role of citrate in modulating gene regulation in *S. aureus*.

The manuscript is extensive and dense, which can sometimes lead to confusion. In my opinion, the authors should consider condensing it.

Response: Thank you for pointing these out. In the revised manuscript, we have carefully condensed and revised the manuscript for improved clarity and readability.

Reviewer #3: Chen, F. and colleagues present a manuscript on the role of citrate as a regulatory molecule modulating carbon metabolism and iron homeostasis.

The authors show that mutant strains of S. aureus with altered intracellular levels of citrate (TCA cycle mutants) have strongly altered metabolic and transcriptional profiles. They show that citrate binds directly to the transcriptional regulator CcpE with diverse regulatory effects. Additionally, it is suggested that the ability of citrate to chelate metals (especially Fe and Mg) alters Fur-activity thereby impacting transcription of iron-acquisitions genes. Furthermore, CcpE and Fur are shown to have overlapping DNA recognition sites, further supporting the role of CcpE (and with it citrate) in modulating iron starvation responses. Finally, the effects of mutants are validated using animal models of infection.

The concept of this study adds a novel layer of complexity of iron starvation responses in S. aureus und is relevant for the readership of this.

This manuscript contains an enormous amount of data and experiments addressing the topic at with various technical approaches and all relevant physiological levels. The authors have to be congratulated to this comprising effort.

It has to be said, that some of the individual experiments are lacking individual controls that would strengthen the drawn conclusions. E.g. Complex formation of CcpE in the presence of citrate (Fig. 2a) using formaldehyde crosslinking is little convincing and appropriate ΔCcpE controls are not available. However, the authors show also crystals of citrate-CcpE complementing the deficit of the other experiment. Similarly, the authors show that Fur does not bind to its DNA target when excess citrate is available (Fig. 5e) claiming it to be caused by metal-chelation by citrate. Appropriate controls including equimolar citrate:metal concentrations are not included. However, in the general context of data showing intracellular metal levels, protein expression levels, transcriptional changes etc. these small flaws do not question the general drawn conclusions.

Response: We are pleased to hear that the reviewer acknowledges that our study contributes a novel layer of complexity of iron starvation responses in *S. aureus*.

We are also thankful to the reviewer for the very valuable and constructive criticism on our study. By incorporating your comments and taking into consideration the comments from Reviewer #1 and #2, we have removed the reference to the "the result of formaldehyde crosslinking experiment" from the revised manuscript. Additionally, we carried out additional control EMSA experiments as suggested by you. The results are presented in Fig. 6E of the revised manuscript, demonstrating that in the presence of excess citrate, Mn^2+^ increase the binding activity of Fur to *isdC* promoter in a dose-dependent manner.

Part II – Major Issues: Key Experiments Required for Acceptance

Please use this section to detail the key new experiments or modifications of existing experiments that should be absolutely required to validate study conclusions.

Generally, there should be no more than 3 such required experiments or major modifications for a "Major Revision" recommendation. If more than 3 experiments are necessary to validate the study conclusions, then you are encouraged to recommend "Reject".

Reviewer #1: Major issues:

1. Some sections are complex and difficult to parse. For instance: Lines 120-125. Please review the entire manuscript for other instances.

Response: Response: Thank you very much for pointing that out. We have modified the sentences as “Among the 678 up-regulated features, 387 were found to be up-regulated upon the deletion of *ccpE* in the ^Tn^*citB* mutant (1D Fig.). Meanwhile, of the 294 down-regulated features, 123 were also found to be down-regulated in the ^Tn^*citB* mutant as a result of the deletion of *ccpE* (1D Fig., Sheet 1 of S1 Table).” (Page 6, Line 135-138 in the revised manuscript).

Additionally, we have thoroughly revised the manuscript in order to make the presentation clear.

2. For differential changes in gene expression and metabolite levels, the phrasing citZ citB vs. citB is vague. Please use citZ citB / citB etc for comparisons to clarify the over- or under-expression ratios.

Response: By following your suggestion, we used ∆*citZ*^Tn^*citB*/^Tn^*citB*, etc., for the comparison in the revised manuscript.

3. Fig 2 and Fig S4: Western blot resolution is poor and not convincing for demonstrating citrate alters oligomerization of CcpE.

Response: By incorporating your comments and taking into consideration the suggestions from Reviewer #2 and #3, we have removed the reference to the "the result of formaldehyde crosslinking experiment" from the revised manuscript.

4. Lines 233-235: The thermal shifts are rather small. Is this typical? Perhaps cite other examples where true binding affects thermal shift in this small amount.

Response: We have added a sentence to the revised manuscript as following (line 206-208): Ligand binding has been reported to cause a relatively low shift in the *Tm* of other proteins as well [23-25].

5. Line 238: D256A variant – control western seems missing that shows that it is made to WT levels.

Response: By following your comments, we have revised the sentence as following (Line 211-213): This substitution had no discernible impact on CcpE abundance in *S. aureus* (S4D and S4E Fig.), similar to the replacement of Arg256 with alanine [12].

6. Lines 324-340: The data for CcpE controlling pycA seems confusing to me. Perhaps it’s the writing, but I don’t understand the model: the ccpE mutant has increased pycA transcript and pycA-lacZ activity, and CcpE addition to in vitro transcription assays blocks transcription. This is all consistent with CcpE as a repressor. However, mutating the T-N11-A motif results in a loss of the upregulation in both WT and ccpE cells.

Response: We apologize for any confusion caused. In our revised manuscript, we have included the following sentence (Line 288-291): Disrupting the T-N_11_-A motif led to a loss of upregulation in both wild-type and Δ*ccpE* cells (4C and 4D Fig.), indicating that the T-N_11_-A motif is not only crucial for the regulation of *pycA* by CcpE but also plays a significant role in the promoter activity of *pycA*.

7. Lines 353-355: this is a nice result, but somewhat indirect. Can you make a stronger conclusion by doing a more direct experiment? For instance, can you uncouple the expression of pycA from its regulation? Maybe express from an inducible or constitutive promoter?

Response: We conducted additional experiments and have included the corresponding results in Figure 4I of the revised manuscript. These results demonstrate that constitutive expression of *pycA* fully restored the growth of ^Tn^*citB* mutant to a level comparable to that of the wild-type Newman strain.

8. CcpE modulates Fur-regulated genes section: On some level this becomes very descriptive, and dense. “To investigate further…” Why? What is a detail and what is important here? This reads as unfocused. Maybe clarify your model here, and design experiments to test specific hypotheses? Why the dual regulation by CcpE and Fur? Do they both use the same binding sites? Or one each? Mutagenesis can help here. Also: “To clarify the influence….” Is there a reason to expect post-transcriptional regulation? Again, isn’t the interesting and big question the mechanism how CcpE and Fur use the same site?

Response: Based on your feedback, we have renamed the section as " CcpE competes with Fur for binding to the intergenic region of *isdA* and *isdC*" and revised the content in the section to improve its clarity and presentation (Page 15, line 316-317).

9. Lines 418-421: The authors use the word “suggest” here, but still seems overreaching. CcpE’s effect is on transcription, as far as we know. Anything else is speculative.

Response: We agree. We have removed the sentence/statement from the revised manuscript.

10. The Citrate accumulation and activation of CcpE section seems like it would be better placed before CcpE modulates iron homeostasis in citB mutant section. I was lost in the latter section until I came to the former section.

Response: By following your suggestion, we have repositioned the section "Citrate accumulation and activation of CcpE are triggered by iron starvation" before the section "CcpE modulates iron homeostasis in ^Tn^*citB* mutant" in the revised manuscript.

11. Animal experiments in Fig 6: there is significant dispersion in the data for some experiments; thus, it’s hard to make firm conclusions.

Response: We sincerely thank you for your attentive reading. While some of the animal infection data did disperse, we have presented the *p*-value determined by the Mann-Whitney test.

In response to your feedback, we have thoroughly revised the section to enhance clarity. For instance, we have replaced the phrase “exhibited significant decreases” as “exhibited statistically significant decreases” (line 426). Additionally, we have refined the statement regarding the bacterial loads of the ^Tn^*citB* mutant in the kidneys, changing it to “increased the bacterial loads of the ^Tn^*citB* mutant in the kidneys in a statistically significant manner”. (line 433-434)

12. What is the purpose of the experiment in 6D? Seems peripheral to the story.

Response: We have omitted the original Figure 6D from the revised manuscript.

13. For as long and expansive the manuscript is, the discussion is relatively shallow and doesn’t really contextualize the data in a meaningful way.

Response: Thank you for your comment. We have addressed this concern by expanding the discussion section to provide a more thorough contextualization of the data in the revised manuscript (Line 478-485, Line 492-494, Line 514-550, Line 552-554).

Reviewer #2: 1. I understand the rationale behind utilizing the citB and citZcitB mutants throughout the manuscript. Nonetheless, it's important to note that both of these mutants exhibit partial impairment of the TCA cycle (as evidenced by post-experimental growth defects in Fig S1B), which introduces complexity into the interpretation of whether observed effects are solely attributable to citrate or influenced by impaired TCA cycle function or growth-related factors.

Response: We express our sincere gratitude for your attentive reading and insightful comments. In response to your feedback, we have added the following sentence into the revised manuscript (Page 22, Line 480-485): “However, it is important to note that both the *citB* and Δ*citZ*^Tn^*citB* mutants exhibit a partial impairment of the TCA cycle (as evidenced by post-experimental growth defects in 1C Fig.), introducing complexity in determining whether the observed effects are solely attributed to citrate or influenced by impaired TCA cycle function or growth-related factors.”

2. Regarding the citB and citZcitB mutants, it is theoretically expected that they should not be capable of producing physiological levels of citrate within cells. In the citB mutant, citrate levels are notably elevated (~16 mM), while in the citZcitB mutant, inactivation of citZ should lead to a complete depletion of the citrate pool, despite the authors reporting citrate levels close to those of the wild type.

a. It would be beneficial if the authors could confirm the intracellular source of citrate in the citZcitB mutant.

Response: By following your suggestions, we have performed additional experiments and the new results are now included in Fig. S1 of the revised manuscript. The corresponding findings are presented in the text from Line 112 to Line 125 and as following: “Unexpectedly, we observed that the intracellular citrate levels of the ∆*citZ*^Tn^*citB* mutant were similar to those of the WT Newman strains (1B Fig.). We hypothesized that SbnG, another citrate synthase in *S. aureus,* might contribute to the intracellular citrate pool in the ∆*citZ*^Tn^*citB* mutant. Deletion of *sbnG* did not significantly affect the intracellular citrate levels of the ∆*citZ*^Tn^*citB* mutant cultured in TSB medium (S1A Fig.). However, when cultured in tryptic soy broth (TSB) containing 1mM iron chelator 2,2′-dipyridyl (DIP), the Δ*sbnG*Δ*citZ* ^Tn^*citB* triple mutant showed an undetectable level of intracellular citrate (S1B Fig.). In contrast, the complemented strain, Δ*sbnG*Δ*citZ* ^Tn^*citB*/p-*sbnG*, exhibited comparable intracellular citrate levels to the ∆*citZ*^Tn^*citB* mutant (S1B Fig.), suggesting that SbnG plays a crucial role in regulating the intracellular citrate levels of the ∆*citZ*^Tn^*citB* mutant under iron deprivation conditions. The exact source of intracellular citrate in the ∆*citZ*^Tn^*citB* mutant cultured in iron-replete TSB medium remains unknown, warranting further investigation to clarify this aspect.”

b. Given that these mutants may not accurately reflect physiological citrate levels, it would be valuable to provide evidence demonstrating that, under physiological concentrations in the wild-type, citrate can indeed function as a signaling molecule to regulate this extensive regulon.

Response: Following your suggestion, we have performed additional experiments and the new results are now included in Fig. 4J of the revised manuscript. The corresponding findings are presented in the text from Line 311 to Line 314 and as following: “Furthermore, we observed that DIP treatment decreased the expression of *pycA* in the wild-type Newman strain, while it failed to do so for the ∆*citZ*, ∆*citZ*∆*sbnG*, or the ∆*ccpE* mutant (4J Fig.), supporting the notion that citrate activates CcpE and that CcpE represses *pycA*.”

3. Although the authors have used omics-based studies, there isn’t a good summarization of the major impact of CcpE on cellular metabolism. For instance, what are the major genes of central and peripheral metabolism that are directly controlled by CcpE? Do the differences in metabolism as found from metabolomics and RNA seq correlate with the direct targets of CcpE? The closest the authors have come to is give a summarization in Fig S6. However, this figure is not sufficiently discussed to give the readers what CcpE does globally in the cell.

Response: Following your kind advice, we have included a further deep discussion based on Fig. S6 according to the omics-based results. These discussion are now presented in the text from Line 514 to Line 550 and as following: “The metabolism of glucose plays a crucial role in the *S. aureus* pathogenicity [9,53]. Studies have indicated that elevated glucose levels in the hyperglycemic abscesses can significantly boost the virulence potential *S. aureus*, leading to more severe infection outcomes [54]. The utilization of glucose by *S. aureus* can occur through two main metabolic pathways: fermentation and respiration [18,55,56]. These pathways appear to play a significant role in determining the levels of citrate within the bacterium [18,55,56]. During fermentation, *S. aureus* metabolizes glucose to produce energy in the absence of oxygen, resulting in the formation of fermentation byproducts such as lactate, ethanol, and acetate [55,56]. On the other hand, when oxygen is present, *S. aureus* can switch to aerobic respiration, fully oxidized glucose through the citric acid cycle and oxidative phosphorylation [18,55,56]. We observed that deletion of *ccpE* in the ^Tn^*citB* mutant led to markedly increased expression of genes, such as *butA*, *NWMN_2459*, *lctP1*, *NWMN_1315*, *NWMN_2026*, *adh1*, *pflB*, and *fdhA*, associated with fermentation pathways [57,58] (S6 Fig.). Additionally, in the pathway from pyruvate to L-leucine through the 2,3-Dihydroxy-3-methylbutanoate in the ^Tn^*citB* mutant, the levels of L-leucine and 2,3-Dihydroxy-3-methylbutanoate were significantly upregulated upon *ccpE* deletion, while glycolytic intermediates like glycerate-3P, glycerate-2P, and phosphoenolpyruvate (PEP) exhibited a decrease in abundance (S6 Fig.). Furthermore, we also observed that level of L-alanine, a fermentation product, was increased in the ^Tn^*citB* mutant by the deletion of *ccpE* (S6 Fig.). These results, combined with CcpE's role as an activator of the TCA cycle [12,28], suggests that CcpE may play a pivotal role in coordinating the metabolic transition from fermentation to respiration.

Interestingly, in addition to *pycA*, CcpE also binds to the promoter regions and inhibits the transcription of *zwf* and *glmS* (S6 Fig.). In bacteria, the protein product of *zwf* diverts glucose-6P from glycolysis into the pentose phosphate pathway, while that of *glmS* catalyzes the conversion of fructose-6P to GlcN-6P [59] (S6 Fig.). These observations are also consistent with the finding that deletion of *ccpE* decreased the levels of glycolytic intermediates like glycerate-3P, glycerate-2P, and phosphoenolpyruvate (PEP) in the ^Tn^*citB* mutant (S6 Fig.), suggesting that activation of CcpE may help to direct the carbon flux to the glycolytic pathway. Moreover, the impact of CcpE on central carbon metabolism may be more profound than previously thought. CcpE can bind to promoter regions and/or influence the expression of genes involved in the formation (or consumption) of almost all 12 branchpoint metabolites (S6 Fig.), which serve as exit points for carbon from the central carbon metabolism network in bacteria [35] (S6 Fig.).”

4. Fig 2A and 2B- The tetramer to monomer ratio determination from immuno blots is not very convincing at all. First, there are no loading controls. Second, the dimer and tetramer bands are barely visible, making me wonder how robust the quantification in 2B is.

Response: By incorporating your comments and taking into consideration the comments from Reviewer #1 and #3, we have removed the reference to the "the result of formaldehyde crosslinking experiment" from the revised manuscript.

5. The finding that CcpE inhibits pycA is intriguing and Fig S7 should really be in the main text. However, one aspect that is not clear to me is why CcpE is an activator for some genes and a repressor for others. Is there a difference in the location of CcpE binding site for activated and repressed genes?

Response: We have now relocated the original Fig. S7 from the supplementary material to the main text of the revised manuscript, where it is now presented as Fig. 4.

We are currently investigating the mechanisms underlying the dual role of CcpE as an activator for some genes and a repressor for others. We are committed to exploring this aspect further in future studies.

6. Ln 127: In Fig 4F, it is unclear what the rationale for adding 1 mM Dipyridyl is in TSB. Also, what is the concentration of iron in Newman strain?

Response: We have conducted an analysis of the iron concentration in the ^Tn^*citB*, ∆*ccpE*^Tn^*citB*, and the ∆*ccpE*^Tn^*citB*/p*-ccpE* strains cultured in iron-replete TSB medium. Although the results of this analysis were not included in the original manuscript, they have now been added to Fig. 6A in the revised manuscript.

The WT Newman strain was excluded from the analysis for economic reasons. We believe that the results of the WT Newman strain are not essential for drawing the conclusion that "CcpE modulates iron homeostasis in the ^Tn^*citB* mutant."

7. Ln 433- 447: The rationale for using NRPMI medium is weak and the narrative on iron homeostasis is lost. Infact, I am not certain that the Hemin experiments add much to the narrative. The Streptonigrin experiments are proof enough that increased intracellular citrate in CitB likely scavenges iron.

Response: Following your feedback regarding the extensive and dense nature of the manuscript, we have omitted the references to the "NRPMI medium" and "Hemin experiments" in the revised version. We sincerely appreciate your valuable input.

8. The fact that Citrate can impact Fur repressor activity by a) directly chelating intracellular iron which is essential for Fur repressor activity and b) by co-activating CcpE which competitively displaces Fur is a strong finding of this study. However, this message is not necessarily coming through in the text, although there is a figure dedicated to this.

Response: Thank you for pointing this out. We have added a sentence to the Discussion section in the revised manuscript as following (Page 22, Line 478-480): ”Additionally, citrate can also influence Fur repressor activity by directly chelating intracellular iron, which is essential for Fur repressor activity [40,49], and by co-activating CcpE, which competitively displaces Fur (5C and 5D Fig.).”

9. Ln 661- The authors state that citrate turns off gluconeogenesis and turns on TCA cycle. This is confusing as active gluconeogenesis is needed to metabolize citrate TCA cycle substrates in the post exponential phase. What is the evidence that gluconeogenesis is impacted when citrate levels are high?

Response: We apologize for any confusion caused. In the revised manuscript, we have modified the sentence to read as follows: “Citrate acts as a key link between multiple cellular processes, coordinating the glycolytic pathway, TCA cycle, and iron uptake at the transcriptional level in *S. aureus* (S6 Fig. and 7D Fig.)” (Line 552-554 in the revised manuscript).

Reviewer #3: My only major concern is that the authors build their experiments solely on mutants of the TCA cycle to alter endogenous citrate levels. Obviously, such mutations are impacting physiological processes dramatically and will result in “sick” strains with altered metabolic fluxes. Exogenous supply of excess nutritional citrate or forcing S. aureus to ferment/respire (glucose vs. glycerol as a carbon source) seem subtler and more physiologically relevant to impact endogenous citrate levels and should be considered to validate phenotypes of the mutant strains.

Response: In response to your feedback, we have performed additional experiments using WT strain and the new results are now included in Fig. 4J of the revised manuscript. The corresponding findings are presented in the text from Line 311 to Line 314 and as following: “Furthermore, we observed that DIP treatment decreased the expression of *pycA* in the wild-type Newman strain, while it failed to do so for the ∆*citZ*, ∆*citZ*∆*sbnG*, or the ∆*ccpE* mutant (4J Fig.), supporting the notion that citrate activates CcpE and that CcpE represses *pycA*.”.

These new findings, in conjunction with other observations in our studies, suggest that alterations in endogenous citrate levels affect the regulatory activity of CcpE in wild-type Newman strain under condition of iron deprivation.Again, we thank you so much for the constructive suggestions in our studies toward the regulatory role of citrate.

Part III – Minor Issues: Editorial and Data Presentation Modifications

Please use this section for editorial suggestions as well as relatively minor modifications of existing data that would enhance clarity.

Reviewer #1: Minor issues:

1. A figure showing the TCA cycle and the genes/enzymes would help the reader evaluate the experiments.

Response: We have incorporated a diagram (Fig. 1A) illustrating the TCA cycle along with the corresponding genes in the revised manuscript.

2. Lines 75-78: Please cite Waters et al. (https://pubmed.ncbi.nlm.nih.gov/27116338/). It gives a more current picture of the role of CodY in regulating metabolism and virulence

Response: The reference (Ref 20) has been included in our revised manuscript.

3. Line 166: I did not see scn in the table. Please check.

Response: We apologize for the discrepancy identified in Tab. S2. The product of scn was initially annotated as SCIN-A, sourced from the aureowiki website (<https://aureowiki.med.uni-greifswald.de/SAUPAN005018000>). To maintain consistency, this annotation has been revised to "SCIN-A/Scn" in S2 Table.

4. Line 169: contract should be contrast

Response: We have made the necessary correction.

5. Lines 184-190: This section requires better rationale/lead-in

Response: We have carefully revised the section for improved clarity and readability.

6. Lines 246-248. Please reference the IDAP-Seq technique: <https://www.pnas.org/doi/full/10.1073/pnas.1300428110>

Response: The reference (Ref 26) has been included in our revised manuscript.

7. Lines 251-255: This is confusing. Please rephrase.

Response: We have revised the sentences as follows: Out of the 390 enriched peaks, 238 (61%) had peak summits located at intergenic regions (Sheet 1 of S4 Table), which constitute only 16.6% of the *S. aureus* Newman genome [27]. The substantial enrichment of CcpE-binding sites in these intergenic regions, where regulatory sites are commonly located, supports CcpE’s role as a transcription regulator. (Line 226-230 in the revised manuscript).

8. Lines 292-294: This may or may not be true. The sites may not be physiologically relevant for a number of reasons. These include incorrect position, and if another regulator binds at the same region and prevents binding.

Response: We have revised the sentences as follows: Upon annotating the 390 CcpE-enriched peaks based on their positions, it was revealed that 682 genes within 443 predicted transcription units (TUs) might potentially be regulated by CcpE (Sheet 1 of S4 Table.). (Line 242-244 in the revised manuscript)

9. Lines 341-343: How does aspartate contribute to NAD synthesis? Please clarify. It is also not clear from the pathway figure.

Response: In response to your feedback, we have modified the statement “In *S. aureus*, PycA supplies oxaloacetate, which serves as a precursor for aspartate, a central building block for many metabolic processes such as biosynthesis of other amino acids (S6 Fig. )[35].” (Line 295-297 in the revised manuscript)

10. Growth curves should be plotted log OD vs time.

Response: The growth curves have now been plotted as per your suggestion in the revised manuscript.

11. Line 474-476: Can this be tested? Seems important to show.

Response: We sincerely appreciate your valuable input. In response to your feedback and reviewer 2's comments on the extensive and dense nature of the manuscript, we have removed the statement, “This could be attributed to the lack of iron incorporation in CitB’s [4Fe-4S] cluster, the only aconitase enzyme in *S. aureus* that depends on this cluster for its enzymatic activity [36, 37] (5C Fig).” in the revised version.

12. Lines 524-527: Why is acetate the control here, when they are not structurally similar? Better justification is required.

Response: Thank you for pointing this out. We have removed the data and the corresponding statement in the revised manuscript.

13. Lines 529-531: I don’t see much difference here…maybe for sirA and sbnA, but not that much. Importantly, why t test and not ANOVA?

Response: We appreciate your thorough review and have revised the statement to read as follows: “Additionally, we observed that the deletion of *citZ* had a greater influence on the expression of Fur-regulated genes, including *sirA* and *sbnA* (6G. Fig.), and to a lesser extent on *isdC* and *isdA* (6H Fig.), in the ^Tn^*citB* mutant in comparison to the *ccpE* deletion.” (Line 413-416)

Additionally, in this set of data, the ANOVA test was utilized instead of the *t*-test.

14. Lines 544-548: CFU/organ induction. Do you mean ~1 log increase in CFUs? Please clarify.

Response: We are very sorry about the confusion. We have revised the statement to read as follows: “Interestingly, we observed that the Δ*citZ*^Tn^*citB* mutant displayed approximately a 0.9-log increase in CFU bacterial loads in the kidneys and hearts of the infected mice compared to the ^Tn^*citB* mutant,”. (Line 428-430)

15. Lines 577-579: It’s a correlation. Nothing more without direct testing.

Response: In the revised version, we have deleted the statement: “This suggests that decreased expression of *pycA*, resulting from the activation of CcpE due to citrate accumulation, may contribute to the virulence attenuation of the ^Tn^*citB* mutant.”

16. G. mellonella experiments are somewhat descriptive. We don’t learn that much more from these experiments compared to what we learn from the CFU burden experiments, other than there is an effect for the JE2 strains. Maybe a better justification is needed for this experiment if it remains in the manuscript.

Response: The results of the *G. mellonella* experiments have been removed from the revised version.

17. Lines 620-622: “…currently less available information…” Not clear from the reference provided.

Response: In the revised manuscript, a new reference (ref. 48) has been cited and the statement was revised as “However, citrate seems to do not affect bacterial PFK [48], and there is currently less available information on the regulatory functions of citrate in prokaryotes cells.” (Line 470-472).

Reviewer #2: 10. Ln 169, correct ‘contract’ to ‘contrast’.

Response: We have made the necessary corrections.

11. Ln 218, remove ‘was’ from this line

Response: We have made the necessary corrections.

12. Ln 339, delete extra ‘of’.

Response: We have made the necessary corrections.

13. Ln 424 replace ‘science’ with ‘since’

Response: We have made the necessary corrections.

Reviewer #3: Discussion.

S. aureus citrate levels are dependent on fermentation vs respiration. The authors use experimental conditions with glucose as the major carbon sources. Accordingly, S. aureus will ferment and the citrate cycle will be shut down giving generally low citrate levels and turnover. The authors should discuss the relevance of their finding in this light and might include thoughts about the role of Ccp in switching metabolism from fermentation to respiration.

The effects of citrate levels on S. aureus metabolism have also been investigated by others, https://pubmed.ncbi.nlm.nih.gov/36472430/. On first glace it appears that observed effects can support the findings of this manuscript.

Results of the in vivo experiments are difficult to interpret. Maybe the finding that isd is poorly accepting mouse Hb can help the interpretation https://pubmed.ncbi.nlm.nih.gov/21147468/.

Response: Following your suggestion, we have included two paragraphs in the discussion section as follows (Line 514-550): “The metabolism of glucose plays a crucial role in the *S. aureus* pathogenicity [9,53]. Studies have indicated that elevated glucose levels in the hyperglycemic abscesses can significantly boost the virulence potential *S. aureus*, leading to more severe infection outcomes [54]. The utilization of glucose by *S. aureus* can occur through two main metabolic pathways: fermentation and respiration [18,55,56]. These pathways appear to play a significant role in determining the levels of citrate within the bacterium [18,55,56]. During fermentation, *S. aureus* metabolizes glucose to produce energy in the absence of oxygen, resulting in the formation of fermentation byproducts such as lactate, ethanol, and acetate [55,56]. On the other hand, when oxygen is present, *S. aureus* can switch to aerobic respiration, fully oxidized glucose through the citric acid cycle and oxidative phosphorylation [18,55,56]. We observed that deletion of *ccpE* in the ^Tn^*citB* mutant led to markedly increased expression of genes, such as *butA*, *NWMN_2459*, *lctP1*, *NWMN_1315*, *NWMN_2026*, *adh1*, *pflB*, and *fdhA*, associated with fermentation pathways [57,58] (S6 Fig.). Additionally, in the pathway from pyruvate to L-leucine through the 2,3-Dihydroxy-3-methylbutanoate in the ^Tn^*citB* mutant, the levels of L-leucine and 2,3-Dihydroxy-3-methylbutanoate were significantly upregulated upon *ccpE* deletion, while glycolytic intermediates like glycerate-3P, glycerate-2P, and phosphoenolpyruvate (PEP) exhibited a decrease in abundance (S6 Fig.). Furthermore, we also observed that level of L-alanine, a fermentation product, was increased in the ^Tn^*citB* mutant by the deletion of *ccpE* (S6 Fig.). These results, combined with CcpE's role as an activator of the TCA cycle [12,28], suggests that CcpE may play a pivotal role in coordinating the metabolic transition from fermentation to respiration.

Interestingly, in addition to *pycA*, CcpE also binds to the promoter regions and inhibits the transcription of *zwf* and *glmS* (S6 Fig.). In bacteria, the protein product of *zwf* diverts glucose-6P from glycolysis into the pentose phosphate pathway, while that of *glmS* catalyzes the conversion of fructose-6P to GlcN-6P [59] (S6 Fig.). These observations are also consistent with the finding that deletion of *ccpE* decreased the levels of glycolytic intermediates like glycerate-3P, glycerate-2P, and phosphoenolpyruvate (PEP) in the ^Tn^*citB* mutant (S6 Fig.), suggesting that activation of CcpE may help to direct the carbon flux to the glycolytic pathway. Moreover, the impact of CcpE on central carbon metabolism may be more profound than previously thought. CcpE can bind to promoter regions and/or influence the expression of genes involved in the formation (or consumption) of almost all 12 branchpoint metabolites (S6 Fig.), which serve as exit points for carbon from the central carbon metabolism network in bacteria [35] (S6 Fig.).” (Line 514-550)

Moreover, in response to your feedback, we have incorporated additional sentences to the results section as follows (Line 446-451): “However, the role of citrate in the pathogenicity of *S. aureus* may be underestimated in the current infection model, as *S. aureus* has developed its Isd system with a heightened capacity to bind hemoglobin derived from humans compared to other mammals [43] and citrate influences the expression of *Isd* genes (5 Fig.). The regulatory effect of citrate or CcpE on the pathogenicity of *S. aureus* seems to be multifaceted, and additional studies are warranted.”

Fig. 1A and D. the dashed and solid arrows in the figure are confusing, reading the text along with the figure, it is not clear what number the authors are rereferring to.

Response: The dashed arrows have been replaced with solid arrows, and the description of this section has been simplified.

Ln. 56 – human

Response: The extra “human” has been deleted.

Ln. 72 - genes involved in

Response: We have made the necessary corrections.

Ln103 – 109: very long sentence that is very difficult to understand. This should be revisd.

Response: We have modified the statement in the revised version.

Ln169 – In contrast

Response: We have made the necessary corrections.

Ln337 – was

Response: The extra “was” has been deleted.

Ln339 – of

Response: The extra “of” has been deleted.

Ln360 – aimed

Response: We corrected it.

Ln406 – Western

Response: The letter “w” has now been capitalized.

Ln408 – 409 - please check if designation of the complementation is really correct here. All complemented individually or together in the same cell?

Response: In the revised manuscript, we have updated the designation to improve clarity.

Ln 394-404 – a diagram showing the organization of the locus and the identified Fur, CcpE binding sites would be helpful to understand this.

Response: We have included a diagram (Fig. 5A) in the revised manuscript as your suggested.

Ln424 – Since

Response: We corrected it.

Ln 647 – TCA derived citrate is only needed for SF-A production not for SF-B production https://pubmed.ncbi.nlm.nih.gov/21147468/

Response: We have incorporated the following statement in the revised manuscript (Line 504-505): "Interestingly, TCA derived citrate is only necessary for staphyloferrin A production, not for staphyloferrin B production [41]."
